# The miR-124-Prolyl Hydroxylase P4HA1-MMP1 axis plays a critical role in prostate cancer progression

**DOI:** 10.18632/oncotarget.2208

**Published:** 2014-07-12

**Authors:** Balabhadrapatruni V. S. K. Chakravarthi, Satya S. Pathi, Moloy T. Goswami, Marcin Cieślik, Heng Zheng, Sivakumar Nallasivam, Subramanyeswara R. Arekapudi, Xiaojun Jing, Javed Siddiqui, Jyoti Athanikar, Shannon L. Carskadon, Robert J. Lonigro, Lakshmi P. Kunju, Arul M. Chinnaiyan, Nallasivam Palanisamy, Sooryanarayana Varambally

**Affiliations:** ^1^ Michigan Center for Translational Pathology, Ann Arbor, MI, USA; ^2^ Department of Pathology, University of Michigan, Ann Arbor, MI, USA; ^3^ Department of Urology, University of Michigan, Ann Arbor, MI, USA; ^4^ Howard Hughes Medical Institute, University of Michigan Medical School, Ann Arbor, MI, USA; ^5^ Comprehensive Cancer Center, University of Michigan Medical School, Ann Arbor, MI, USA; ^6^ Present Address: Department of Hematology and Oncology, Providence Hospital and Medical Center, Southfield, MI, USA

**Keywords:** Prolyl 4-hydroxylase, alpha polypeptide I, Prostate Cancer, Progression, Metastasis, MicroRNA, Matrix metalloprotease 1

## Abstract

Collagen prolyl hydroxylases (C-P4HAs) are a family of enzymes involved in collagen biogenesis. One of the isoforms of P4HA, Prolyl 4-hydroxylase, alpha polypeptide I (P4HA1), catalyzes the formation of 4-hydroxyproline that is essential for the proper three-dimensional folding of newly synthesized procollagen chains. Here, we show the overexpression of *P4HA1* in aggressive prostate cancer. Immunohistochemical analysis using tissue microarray demonstrated that P4HA1 expression was correlated with prostate cancer progression. Using *in vitro* studies, we showed that P4HA1 plays a critical role in prostate cancer cell growth and tumor progression. Expression profiling studies using P4HA1-modulated prostate cells suggested regulation of Matrix metalloprotease 1. The invasive properties of P4HA1 overexpressing cells were reversed by blocking MMP1. Our studies indicate *P4HA1* copy number gain in a subset of metastatic prostate tumors and its expression is also regulated by microRNA-124. MiR-124 in turn is negatively regulated by transcriptional repressors EZH2 and CtBP1, both of which are overexpressed in aggressive prostate cancer. Chick chorioallantoic membrane (CAM) assay and mice xenograft investigations show that P4HA1 is required for tumor growth and metastasis *in vivo*. Our observations suggest that P4HA1 plays a critical role in prostate cancer progression and could serve as a viable therapeutic target.

## INTRODUCTION

Prostate cancer is the most common malignancy and the second most common cause of cancer death among men in the United States [1]. While multiple molecular events characterize prostate cancer initiation, growth, invasion, and metastasis, the exact mechanism of tumorigenesis remains unclear. Thus, identification of oncogenic drivers and potential therapeutic targets is critical for both early diagnosis and effective treatment. Gene expression profiling studies and high throughput transcriptome sequence analyses have revealed tumor-specific gene signatures and multiple oncogenic drivers [2-10]. In this study, we characterized prolyl 4-hydroxylase, alpha polypeptide I (P4HA1) as overexpressed specifically in aggressive prostate cancer. P4HA1 is a key enzyme in collagen biogenesis. The proper triple helical collagen formation involves extensive post-translational modifications including hydroxylation of prolyl and lysyl residues [11, 12]. P4HA1 catalyzes the formation of 4-hydroxyproline that is essential for the proper three-dimensional folding of newly synthesized procollagen chains.

Hypoxia, an essential feature of the neoplastic microenvironment, is known to play a critical role in regulating collagen biosynthesis and regulating P4HAs. Hypoxia also induces hypoxia-stabilized HIF1α (Hypoxia inducible factor-1α) protein that promotes tumor growth, angiogenesis, and metastasis [13]. In hypoxic fibroblasts HIF-1 induces extracellular matrix (ECM) remodeling by activating expression of P4HA1, P4HA2 and PLOD2 leading to changes in cancer cell morphology, adhesion and motility that promote invasion and metastasis [14]. Prolyl 4-hydroxylases are shown to play a role in breast cancer metastasis and serve as prognostic marker [15].

In this study, we present evidence suggesting that overexpression of P4HA1 plays critical role in prostate cancer progression. In a subset of prostate cancer we found copy number gain of P4HA1. Our knockdown studies demonstrated that P4HA1 expression is required for prostate cancer cell proliferation and invasion. In addition, we showed that *P4HA1* is a miR-124 target gene. MiR-124 in turn is regulated by transcriptional repressor Enhancer of Zeste Homolog 2 (Drosophila) EZH2 and transcriptional co-repressor C-terminal binding protein 1 (CtBP1), genes that are overexpressed in aggressive prostate cancer [7, 16]. Furthermore, mouse xenograft studies demonstrated a role for P4HA1 in tumor growth *in vivo*. We also observed altered expression of Matrix metalloproteases (MMP1 and MMP2) and Fibronectin leucine rich transmembrane protein 3 (FLRT3) upon P4HA1 perturbation in cancer cells. With multiple MMP1 inhibitors in clinical trials, MMP1 could potentially serve as a surrogate target and benefit the prostate cancer patients that overexpress P4HA1. Our studies provide a rationale for targeting P4HA1 in aggressive prostate cancer.

## RESULTS

### Prolyl hydroxylase P4HA1 is overexpressed in aggressive prostate cancer and predicts disease progression

**Figure 1:**
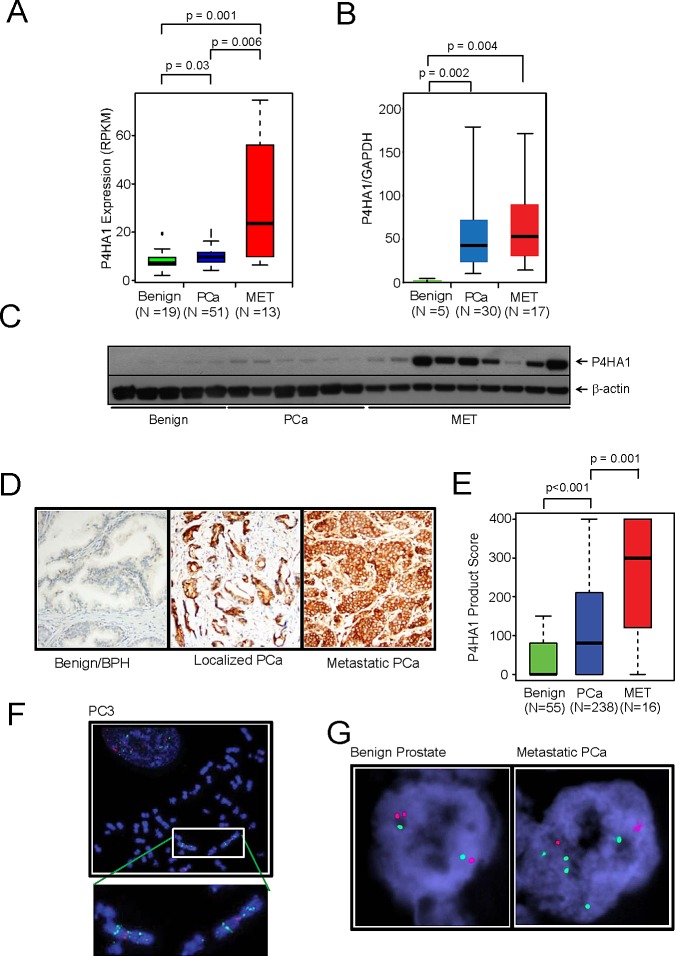
Collagen prolyl hydroxylase P4HA1 is overexpressed in prostate cancer and is associated with disease progression A, *P4HA1* gene expression from NGS data and B, quantitative real-time PCR of RNA from benign, prostate carcinoma (PCa) and metastatic prostate cancer (MET) tissues. C, P4HA1 protein expression by immunoblot analysis of prostate tissue extracts using P4HA1 antibody. β-actin was used as a loading control. D, Immunohistochemical analysis of P4HA1 in benign prostate epithelia (left), primary PCa (center) and metastatic PCa (right). E, P4HA1 staining intensity product scores for benign, primary prostate cancer and metastatic prostate cancer. A progression box plot representing the mean ± SEM values of P4HA1 protein expression in the tissue microarray for each of the indicated groups. P4HA1 expression progressively increases with the severity of the disease (p=0.001). F, Fluorescent *in situ* hybridization (FISH) using *P4HA1* locus specific probe in PC3 cells showing 5-10 copies of *P4HA1* on the isochromosome 10q. G, FISH analysis showing 4-5 copies of *P4HA1* (green) in a metastatic prostate cancer sample (right) compared to a normal prostate sample with two copies of *P4HA1* and control probe (red, left). See also Supplementary Figure S1.

Gene expression profiling studies and transcriptome sequence analysis showed up-regulation of P4HA1 in metastatic prostate cancer (Figure 1A) [17-19]. In order to validate this observation, we performed real-time qPCR using RNA from multiple prostate cancer and benign tissue samples. Real-time qPCR analysis confirmed the overexpression of *P4HA1* in metastatic prostate cancer tissues relative to benign prostate samples (Figure 1B) as did immunoblot analysis using P4HA1-specific antibody (Figure 1C). We conducted Oncomine Platform (Life Technologies, Ann Arbor, MI) database analyses on publicly available microarray datasets and found that *P4HA1* is over-expressed in prostate adenocarcinoma (Supplementary Fig. S1A; p=8.57E-4) and metastatic samples (Supplementary Fig. S1B; p=2.22E-7) compared with normal tissues [20, 21]. Similarly, elevated levels of P4HA1 protein was observed in metastatic prostate cancer cell lines relative to benign cell lines (Supplementary Fig. S1C). However, *P4HA2* mRNA expression levels were relatively lower than *P4HA1* in malignant prostate cancer tissues and cell lines (Supplementary Fig. S1D, E). Moreover, no appreciable difference was observed in *P4HA2* levels between benign and metastatic tissues and cell lines (Supplementary Fig. S1D, E), suggesting non-overlapping functions between the two isoforms. We investigated the expression of P4HA1 protein in large number of prostate cancer samples by immunohistochemical (IHC) analysis that showed weak or no reactivity in benign tissues but strong staining in the aggressive prostate cancer tissue and metastatic prostate tumors (Figure 1D). Statistical analysis of the tissue microarray IHC analysis suggested a significant progressive increase in P4HA1 expression with disease progression (p=0.001) (Figure 1E). Fluorescence *in situ* hybridization using *P4HA1* locus specific FISH probe revealed copy number gain in aggressive prostate cancer cell line PC3 (Figure 1F). Similarly, a small subset of metastatic prostate cancer tissues were found to have copy number gains of *P4HA1* (Figure 1G, right panel).

### P4HA1 plays an essential role in prostate cancer cell proliferation and invasion

**Figure 2:**
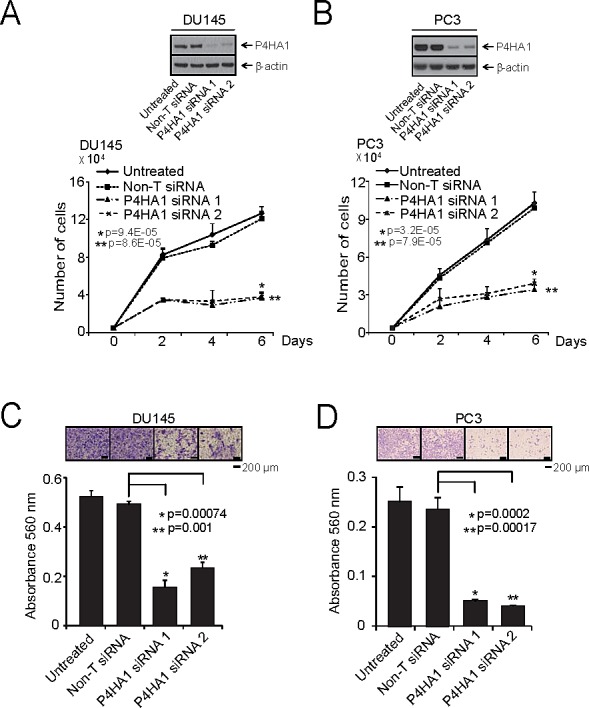
P4HA1 is essential for prostate cancer cell proliferation and invasion A and B, Transient knockdown of P4HA1 in prostate cancer cell lines reduces prostate cancer cell proliferation. Immunoblot analysis of protein using lysates from aggressive prostate cancer cell lines DU145 and PC3 treated with two specific and independent P4HA1 siRNA duplexes. β-actin was used as a loading control. Cell proliferation assay of DU145 and PC3 cells transfected with either P4HA1 siRNA duplex or non-targeting siRNA (Non-T siRNA) control. C and D, Knockdown of P4HA1 reduces DU145 and PC3 cell invasion. Boyden chamber matrigel invasion assay of DU145 or PC3 cells where P4HA1 was transiently knocked down using two independent P4HA1 siRNA duplexes. Non-T siRNA treated cells served as control. Invaded cells were stained with crystal violet and the absorbance was measured at 560 nm. All bar graphs are shown with ± SEM. See also Supplementary Figures S2 and S3.

To determine the functional significance of P4HA1 overexpression in prostate cancer we perturbed P4HA1 levels in prostate cells and tested them in cell proliferation, migration and invasion assays. We utilized both transient RNA interference and stable knockdown strategies targeting P4HA1 in aggressive prostate cancer cell lines, DU145 and PC3. The efficiency of P4HA1 knockdowns were confirmed by immunoblot (Figure 2A, B; Supplementary Fig. S2A) and qPCR (Supplementary Fig. S2B; Supplementary Fig. S3) analyses. We observed significant decrease in cell proliferation upon transient or stable knockdown of P4HA1 compared to control cells transfected with non-targeting si/sh RNAs (Figure 2A, B; Supplementary Fig. S2C, D, respectively). Next, we tested cell motility after stable P4HA1 knockdown in prostate cancer cells using wound healing assay. P4HA1 knockdown showed a wider wound area 24 hours post-wound generation relative to control cells, the delayed time to heal indicating an inability of P4HA1 knockdown cells to migrate (Supplementary Fig. S2E, F). Additionally, P4HA1 knockdown in DU145 and PC3 reduced the invasive potential of these cells as assessed by Boyden chamber matrigel invasion assay (Figure 2C, D). Together, these observations demonstrate the involvement of P4HA1 in the proliferation, migration and invasion of prostate cancer cells *in vitro*.

### MicroRNA miR-124 targets P4HA1 and is down regulated in prostate cancer

**Figure 3:**
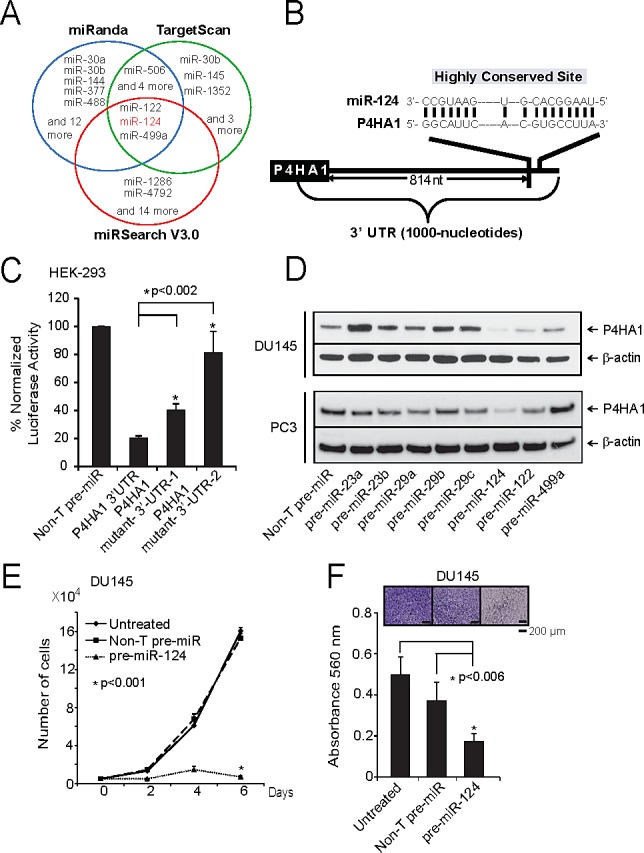
miR-124 targets and down-regulates P4HA1 A, Integrated prediction algorithms displayed as a Venn diagram showing miRNAs computationally predicted to target P4HA1 including miR-124. B, The predicted miR-124 binding sites in the 3′-UTRs of P4HA1. C, Luciferase reporter assay of P4HA-3′-UTR. HEK-293 cells were transfected either with pre-miR-124 or non-targeting pre-miR (NT-pre-miR) along with either P4HA1-3′UTR wild-type, mutant-1 or mutant-2 luciferase constructs. pRL-TK vector was used as an internal control. D, Immunoblot analysis showing P4HA1 protein expression in DU145 and PC3 cells treated with a panel of miRNAs. E, miR-124 reduces prostate cancer cell proliferation. Cell proliferation was measured in DU145 cells ectopically over-expressing either pre-miR-124 or NT-pre-miR. F, Pre-miR-124-treated DU145 cells showed reduced invasion as assessed by Boyden chamber matrigel invasion assay. Invaded cells were stained with crystal violet and the absorbance was measured at 560 nm. All bar graphs are shown with ± SEM. See also Supplementary Figure S4.

Several miRNAs (miRs) act as tumor suppressors by targeting multiple oncogenes leading to their repression. In order to determine if P4HA1 is subject to regulation by miRs in prostate cancer, we utilized microRNA target prediction software programs TargetScan [22], miRanda [23] and miRSearch V3.0 [24]. After integrating the results, we identified 3 common miRs that could potentially target P4HA1, namely miR-122, 124 and 499a (Figure 3A). Since miR-124 had earlier been implicated as tumor-suppressor, we sought to determine its role in P4HA1 regulation by 3′-UTR luciferase assay. The binding site for miR-124 at 3′-UTR of P4HA1 is depicted (Figure 3B). HEK-293 cells co-transfected with miR-124 and pMir-REPORT-P4HA1 3′-UTR plasmids showed substantial reduction in luciferase reporter activity compared to non-targeting control miR (Figure 3C). This effect is reversed by mutating miR-124 target site (Supplementary Fig. S4A, B; Figure 3C). These results indicate that P4HA1 is a direct target of miR-124.

Next, to determine whether miR-124 represses P4HA1 expression, we treated prostate cancer cells with precursor miRs, miR-124 as well as other miRs such as miR-23a, 23b, 29a, 29b, 29c, 122, and 499a and measured P4HA1 protein levels. As shown in Figure 3D, miR-124-treated cell showed significant reduction in P4HA1 protein level, while the control miR precursors did not alter the P4HA1 expression. qPCR analysis in prostate cancer cell lines showed that benign prostate epithelial cell line PrEC expressed greater amounts of miR-124 and lower P4HA1 levels in contrast to aggressive prostate cancer cell lines DU145 and PC3 that express lower miR-124 and higher *P4HA1* levels (Supplementary Fig. S4C). Consistent with the results from cancer cell lines, metastatic prostate cancer tissue samples also expressed low miR-124 and high *P4HA1* mRNA compared to benign samples (Supplementary Fig. S4D). Based on these results we hypothesized that miR-124 acts as tumor suppressor in prostate cancer. We next examined the functional role of miR-124 using DU145 and PC3 cancer cells that were transiently transfected with miR-124 precursor. MiR124 expression dramatically inhibited proliferation of DU145 and PC3 cells compared to control non-targeting miR (Figure 3E; Supplementary Fig. S4E). Similarly, we observed decrease in cell proliferation in androgen-dependent LnCaP cells, suggesting a broad role for miR-124 and its target P4HA1 in both castration-resistant and hormone-sensitive prostate cancer cells (Supplementary Fig. S4F). Furthermore, ectopic over-expression of miR-124 in DU145 (Figure 3F) and PC3 cells (Supplementary Fig. S4G) significantly reduced the ability of these cells to invade through matrigel compared to the cells expressing control non-targeting miR. Our data suggest that miR-124 targets P4HA1 and acts as tumor suppressor miR in prostate cancer.

### Hypoxia inducible factor HIF1α regulates P4HA1 by regulating miR-124

**Figure 4:**
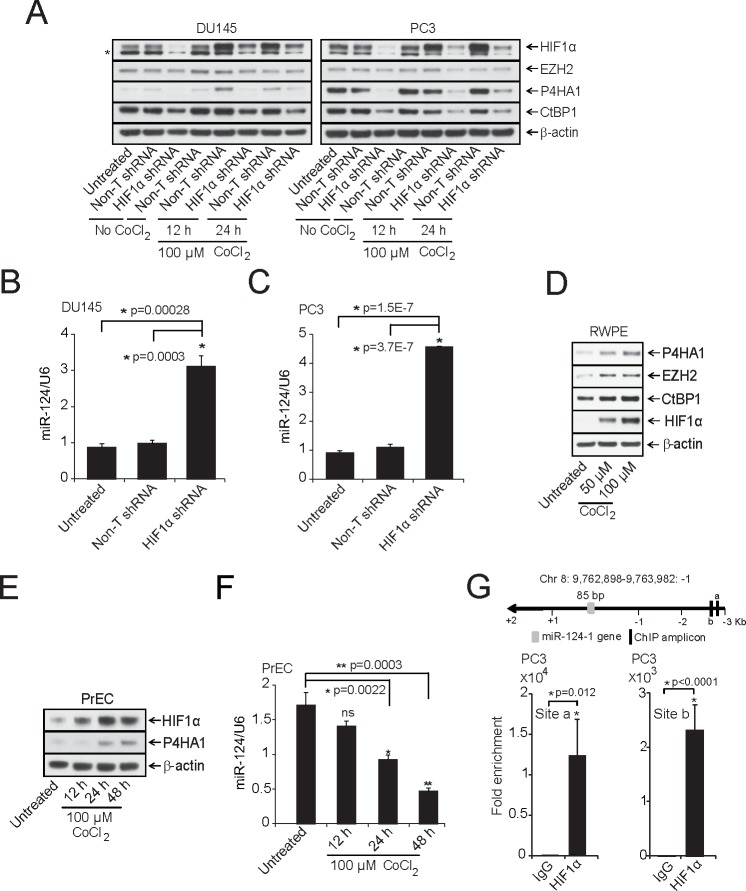
HIF1α modulates P4HA1 expression by down-regulating miR-124 A, Immunoblot analysis of HIF1α -DU145 and -PC3 knockdown lysates under normoxia or in the presence of the hypoxia-mimetic agent CoCl_2_. HIF1α knockdown significantly reduced P4HA1, CtBP1 and EZH2. β-actin was used as a loading control and * denotes an additional band detected by the antibody. B and C, qPCR analysis of miR-124 in HIF1α-stable knockdown DU145 and PC3 cells. D, Benign prostate cancer cell line RWPE was treated with indicated concentrations of CoCl_2_ for 12 h and HIF1α, CtBP1, EZH2 and P4HA1 protein levels were measured by immunoblot analysis. E, Immunoblot analysis of HIF1α and P4HA1 in CoCl_2_-treated normal prostate cell line PrEC. F, miR-124 is down-regulated under hypoxia-mimicking conditions. qPCR analysis of miR-124 in samples from (E); ns, not significant. G, Conventional Chromatin immunoprecipitation (ChIP)-PCR analysis for the HIF1α occupancy on miR-124-1 promoter in PC3 cells following induction with 100 μM CoCl_2_ for 12 h. All bar graphs are shown with ± SEM. See also Supplementary Figures S5 – S7.

To identify upstream regulators of P4HA1, we analyzed the *P4HA1* promoter sequences using Genomatix MatInspector and found several transcription factor binding sites including HIF1α that is known to transactivate P4HA1 [14, 25]. *P4HA1* promoter sequence contains multiple HIF1α binding *hypoxia-response elements* (HRE) “C(G/A)(T/G)G” sites. In addition to inducing protein coding gene expression, hypoxia is known to modulate expression of several miRNAs [26-29]. Here we investigated the effect of HIF1α on P4HA1 and miR-124 expression. In addition, Genomatix MatInspector analysis showed that transcriptional repressor EZH2 and co-repressor CtBP1, both of which are overexpressed in prostate cancer, contain HIF1α binding sites at their promoters. Thus, we also investigated the role of HIF1α in regulating these transcriptional repressors. We performed knockdown of HIF1α in DU145 and PC3 cells and treated these cells with CoCl_2_ to induce hypoxic conditions and performed immunoblot analysis. Knockdown of HIF1α significantly reduced P4HA1 and CtBP1 and moderately EZH2 protein levels, indicating HIF1α role in transactivation of these genes (Figure 4A). Moreover, miR-124 levels were increased in these samples as assessed by qPCR (Figure 4B, C). To further investigate the HIF1α-P4HA1 axis, we incubated benign prostate RWPE cells under hypoxic conditions in the presence of CoCl_2,_ a chemical inhibitor of HIF1α degradation that leads to HIF1α protein accumulation [30]. As expected, hypoxia increased the expression of HIF1α, P4HA1, CtBP1 and EZH2 as shown by immunoblot (Figure 4D) and qPCR analysis (Supplementary Fig. S5A), and also significantly repressed miR-124 levels (Supplementary Fig. S5B (p=0.0004)). Consistent with Cao et al.,[26], we also observed a down-regulation of miR-101 in these samples (Supplementary Fig. S5B). In normal prostate epithelial PrEC cells, we observed concomitant induction of HIF1α and P4HA1 protein levels in the presence CoCl_2_ (Figure 4E). Similar to RWPE, PrEC cells showed lower levels of miR-124 in hypoxic condition (Figure 4F).

To determine whether HIF1α, apart from directly regulating P4HA1, also regulates miR-124, we performed chromatin immunoprecipitation (ChIP) assays with antibody against HIF1α in PC3 and RWPE cells following treatment with 100 μM CoCl_2_ for 12 h. The schematic showing *P4HA1, GLUT-1*(Glucose transporter 1) and *VEGFA* (Vascular endothelial growth factor A) chromosomal loci and ChIP amplicons is depicted in Supplementary Fig. S6A. ChIP-qPCR assay showed strong enrichment of *P4HA1* promoter regions (between 8 × 10^3^ – 5 × 10^4^- fold) by HIF1α antibody relative to IgG control (Supplementary Fig. S6B, C) as did other known targets of HIF1α like *GLUT-1* (Supplementary Fig. S6B, C) and *VEGFA* (Supplementary Fig. S6B, C) that were used a positive controls [31]. Furthermore, we observed HREs in miR-124-1,-2 and -3 promoters. Our ChIP-qPCR assay using miR promoter-specific primers demonstrated strong enrichment of HIF1α at *miR-124-1*, *miR-124-2* and *miR-124-3* promoters in PC3 and RWPE cells (Figure 4G; Supplementary Fig. S7A-C). Thus, our study indicates a dual role of HIF1α in regulating P4HA1 expression both as a transactivator and indirectly by acting as a repressor of miR-124 expression.

### Transcriptional repressors EZH2 and CtBP1 regulate miR-124 expression

**Figure 5:**
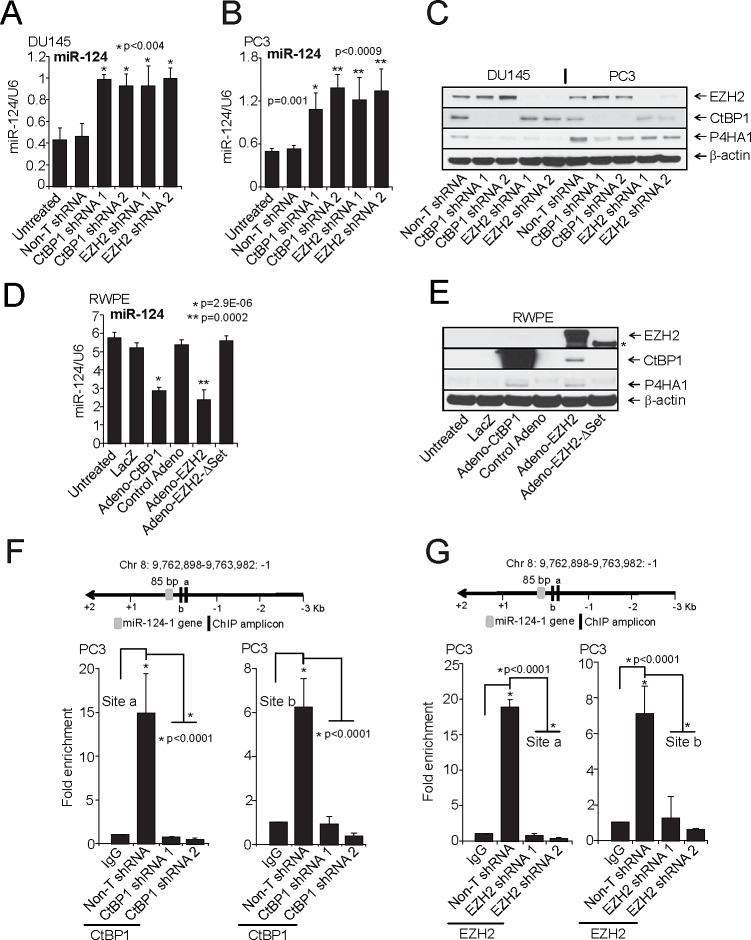
CtBP1 and EZH2 maintain P4HA1 expression by down-regulating miR-124 A and B, qPCR analysis of miR-124 and C, Immunoblot analysis of P4HA1 in CtBP1 and EZH2 stable knockdown DU145 and PC3 cells. D, qPCR analysis of miR-124 and E, Immunoblot analysis of P4HA1, CtBP1 and EZH2 in RWPE cells following infection with control, lacZ adenovirus or CtBP1, EZH2 or EZH2ΔSET mutant adenovirus for 48 h (Asterisk indicates truncated (EZH2^SET^)). F, Conventional Chromatin immunoprecipitation (ChIP)-PCR analysis for the CtBP1 and G, EZH2 occupancy on *miR-124-1* promoter in PC3 cells. ChIP was performed in PC3 Non-T shRNA and respective knockdown cells. ChIP was performed using antibodies against CtBP1, EZH2 and a control IgG. Inset: Schematic representation of the *miR-124-1* genomic region on chromosome 8 showing gene and amplicon positions. Error bars: n = 3. All bar graphs are shown with ± SEM. See also Supplementary Figures S8-S10.

Previous studies demonstrated that miR-124 is down-regulated by epigenetic mechanisms, including DNA methylation and histone modification in various cancers [32-35]. Our previous work suggested EZH2-mediated down-regulation of multiple tumor suppressor miRs such as miR-26, 31, 181a, 181b, 200b, 200c and 203 [36, 37] in prostate and breast cancer. We had also shown that transcriptional co-repressor CtBP1 plays a role in prostate cancer progression by down regulating multiple tumor suppressor genes [16]. We hypothesized that EZH2 and CtBP1 may regulate P4HA1 expression by repressing miR-124. We observed an up-regulation of miR-124 in DU145 and PC3 cells upon stable knockdown of CtBP1 and EZH2 (Figures 5A, B) and a decrease in *P4HA1* mRNA (Supplementary Fig. S8A, B) and protein (Figure 5C). Overexpression of CtBP1 and EZH2 resulted in repression of miR-124 (Figure 5D) and a concomitant increase in *P4HA1* transcript (Supplementary Fig. S8C) and protein (Figure 5E). SET domain mutant EZH2 (EZH2ΔSET) or control adenoviruses did not repress the miR-124 expression. These data support the roles of CtBP1 and EZH2 in maintaining P4HA1 expression by down-regulating miR-124. Our study is corroborated through Oncomine co-expression analysis that shows CtBP1, EZH2 and P4HA1 are co- and over-expressed in prostate carcinoma samples (Supplementary Fig. S8D).

EZH2 is a histone methyltransferase that tri-methylates histone H3 at lysine 27 and represses target gene expression. To demonstrate that CtBP1 and EZH2 target the *miR-124* promoter region, we performed ChIP assays with anti-CtBP1, EZH2 and H3K27me3 antibodies (a mark of EZH2-mediated trimethylation of histone H3 on lysine 27) in DU145 and PC3 cells. As expected, CtBP1 (Figure 5F; Supplementary Fig. S9A-C), EZH2 (Figure 5G; Supplementary Fig. S9A-C) and H3K27me3 (Supplementary Fig. S10A-D) showed enrichment at *miR-124-1* as well as at *miR-124-2* and *miR-124-3* promoter regions. These data suggest that miR-124 is transcriptionally repressed in prostate cancer.

### P4HA1 modulates MMP1 and FLRT3 expression

**Figure 6:**
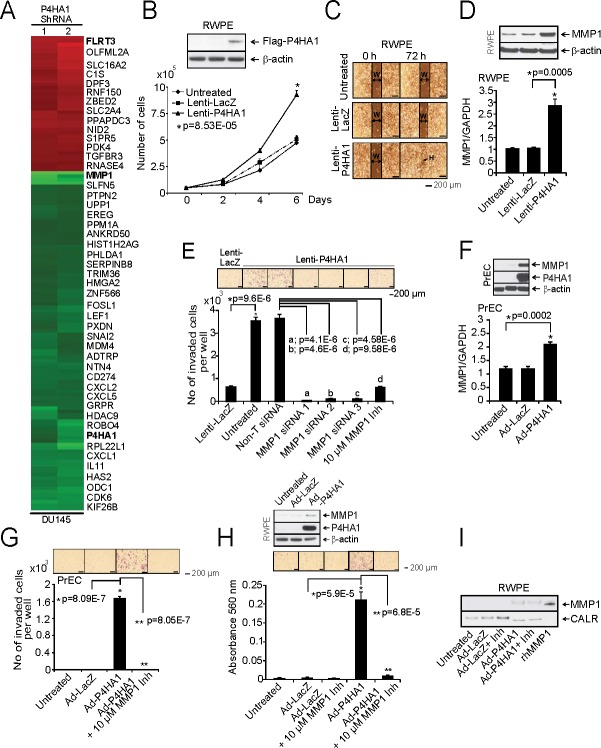
Over-expression of P4HA1 increases cell proliferation and invasion A, Microarray data of selected genes in stable P4HA1 knockdown in DU145 cells. B, Immunoblot analysis showing P4HA1 in RWPE cells (Inset). Stable RWPE-P4HA1 over-expressing cells showed increased cell proliferation than untreated or lacZ over-expressing cells. C, Wound healing assay in stable RWPE-P4HA1 over-expressing cells. An artificial wound was created using a 0.2 ml pipette tip on a confluent monolayer of cells. Images were taken at 0 and 72 h after scratching. The black lines show the margin of scratched area in which double headed arrow indicates scratch width (W) and black arrow indicates complete healing (H) of scratch wound. D, Immunoblot and qPCR analysis of MMP1 in RWPE cells over-expressing lacZ and P4HA1. E, Matrigel invasion assay was performed using lenti-lacZ or lenti-P4HA1 cells. Untreated cells, Non-T siRNA, three independent MMP1 specific siRNA treated cells or in the presence of MMP1 inhibitor were also used in the invasion assay and the invaded cells were counted. Inset, photomicrographs of invaded cells. F, Immunoblot analysis of PrEC cells expressing P4HA1 and qPCR analysis of MMP1 in PrEC cells that are uninfected or lacZ and adeno-P4HA1 infected. G, Matrigel invasion assay using uninfected PrEC cells or cells that are infected with lacZ, adeno-P4HA1 alone or in the presence of 10 μM MMP1 inhibitor. Inset, photomicrographs of invaded cells and H, Matrigel invasion assay using parental RWPE cells or lacZ, adeno-P4HA1 infected cells and adeno-P4HA1 infected cells in the presence of 10 μM MMP1 inhibitor. Inset, photomicrographs of invaded cells and immunoblot analysis of P4HA1. I, Immunoblot analysis of the secreted MMP1 from RWPE cells transiently over-expressing lacZ or P4HA1. The recombinant MMP1 (rhMMP1) served as positive control for MMP1 and CALR is used as a loading control. All bar graphs are shown with ± SEM. See also Supplementary Figures S11 -14.

In order to evaluate P4HA1-mediated effects in prostate cancer progression, we performed gene expression analysis using RNA from P4HA1 knockdown prostate cell lines. We identified multiple genes that were induced or repressed upon P4HA1 knockdown including those involved in tumor growth and invasion such as MMP1, MMP2 and FLRT3, among others (Figure 6A). We validated the activation of FLRT3 and the down-regulation of MMP1 both at mRNA and protein level upon P4HA1 stable (Supplementary Fig. S11A,) and transient (Supplementary Fig. S12A,) knockdown. MMP1 and FLRT3 are known to play a role in metastasis, cell de-adhesion and wound healing [38, 39]. To test the role of FLRT3 in cell migration, we performed a phenotype rescue experiment where we knocked down FLRT3 in DU145-P4HA1 stable knockdown cell lines using two independent duplexes targeting FLRT3 (Supplementary Fig. S11C). FLRT3 knockdown cells healed the gaps faster than in control and P4HA1 stable knockdowns in wound healing assay indicating a critical role for FLRT3 in P4HA1-mediated cell migration (Supplementary Fig. S11D).

Next, we generated stable P4HA1 expressing RWPE cells using lentivirus (Figure 6B, Inset; Supplementary Fig. S13A). Overexpression of P4HA1 resulted in increased cell proliferation (Figure 6B) and wound healing (Figure 6C) confirming the critical role of P4HA1 in prostate cell proliferation. Furthermore, P4HA1 overexpression resulted in increased MMP1 protein expression and reduction in *FLRT3* levels in these cells (Figure 6D; Supplementary Fig. S13A, respectively). To investigate the role of MMP1 in P4HA1-mediated invasion, we treated stable P4HA1 expressing RWPE cells with MMP1 siRNA or MMP1 inhibitor FN-439 [40, 41] and analyzed P4HA1 and MMP1 levels (Supplementary Fig. S13B). Both MMP1 knockdown and MMP1 inhibitor reduced the invasiveness of P4HA1 expressing cells as demonstrated by Boyden chamber matrigel invasion assay (Figure 6E). Similarly, transient overexpression of P4HA1 in normal prostate epithelial cells PrEC as well as RWPE cells with adenovirus increased MMP1 levels and invasion that could also be inhibited by MMP1 inhibitor (Figure 6F-H; Supplementary Fig. S13C, D). While PrEC-P4HA1 and RWPE-P4HA1 cells showed increased invasion, MMP1 inhibitor (10 μM) attenuated the invasion of these cells (Figure 6G, H, respectively). Apart from positive regulation of MMP1 by P4HA1, it also reduced *FLRT3* expression (Supplementary Fig. S13C, D). Our immunoblot analysis suggested that apart from increased cellular expression of MMP1, cell culture supernatant from these adeno-P4HA1 over-expressing RWPE cells contained secreted form of MMP1 while untreated or lacZ expressing cells had no detectable level of secreted MMP1 (Figure 6I).

We next determined expression levels of *MMP1* and *FLRT3* in prostate tissue utilizing transcriptome sequencing data in prostate cancer (Supplementary Fig. S14A, B). Furthermore, we observed an inverse correlation of *FLRT3* and direct correlation of *MMP1* with *P4HA1* in Oncomine co-expression analysis (Supplementary Fig. S14C) [17, 42]. We validated the FLRT3 results across benign, prostrate carcinoma and metastatic prostate cancer tissues by immunoblot analysis (Supplementary Fig. S14D) which confirmed the inverse correlation between P4HA1 and FLRT3. Thus, these data suggest a role for MMP1 and FLRT3 in P4HA1-mediated prostate cancer cell migration and invasion.

### P4HA1 plays a role in prostate tumor growth and metastasis

**Figure 7:**
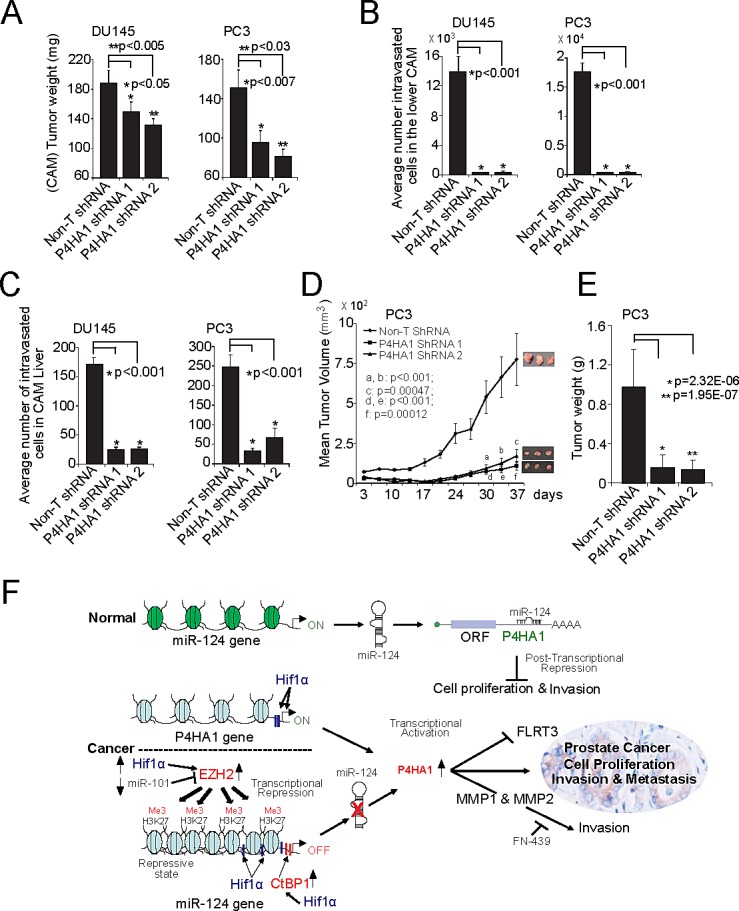
P4HA1 knockdown reduces prostate tumor growth *in vivo* A, Tumor growth of DU145 and PC3 P4HA1 stable knockdown cells or control Non-T shRNA cells in the chick chorioallantoic membrane (CAM) tumor assay. Extra-embryonic tumors were harvested and weights were measured after 72 h of post-implantation of cells. B and C, P4HA1 knockdown reduces metastasis of DU145 and PC3 cells in the CAM models. Metastasized cells to the lower CAM and liver of chicken embryos were quantified using human Alu specific PCR. D, P4HA1 knockdown in PC3 cells inhibits tumor growth in a mouse xenograft model. Plot of mean tumor volume at indicated time points for mice inoculated with Non-T shRNA (solid line with filled diamonds) or two independent P4HA1 stable knockdown shRNA 1 (solid line with filled squares) and 2 (solid line with filled triangles) cells. E, Tumor weights of corresponding mouse xenograft models. n = 8 mice per group. All bar graphs are shown with ± SEM. F, Proposed model of P4HA1, HIF1α and miR-124 regulatory axis in prostate cancer progression. CtBP1, EZH2 or HIF-1α induces P4HA1 expression and maintains its expression by down-regulating miR-124. P4HA1 induces cell proliferation, invasion and metastasis via MMP1 and FLRT3. All bar graphs are shown with ± SEM.

To assess the role of P4HA1 on tumor growth *in vivo*, we employed a chick chorioallantoic membrane assay (CAM) and measured spontaneous metastasis, including local invasion, intravasation, and metastasis to distant organs. CAM was performed as described previously [16] using P4HA1 knockdown DU145 and PC3 prostate cancer cells. Depletion of P4HA1 resulted in significantly reduced tumor weight compared to non-target shRNA-transfected control cells (Figure 7A). P4HA1 knockdown of DU145 and PC3 cells impaired their ability to invade the CAM basement membrane and resulted in significantly decreased number of intravasated cells in the lower CAM compared to control cells (Figure 7B). Furthermore, livers of chicken embryos displayed attenuated metastasis in the P4HA1 knockdown group compared to the control group (Figure 7C). Next, we examined P4HA1-mediated tumorigenesis in a murine PC3 xenograft model using non-targeting shRNA or two independent P4HA1 stable knockdown PC3 cells. Both P4HA1-shRNA 1 and 2 cells showed significantly reduced tumor growth and weight in mice (Figure 7D, E) relative to control animals demonstrating that P4HA1 inhibition attenuates tumor growth and metastasis *in vivo*. These observations clearly suggest that P4HA1 plays a role in prostate tumor growth *in vivo*.

## DISCUSSION

Recent developments in high throughput technologies have paved the way for the identification of new cancer biomarkers for screening and prognostication. In the present study, we investigated the expression and role of prolyl hydroxylase P4HA1 in prostate cancer. Our study showed that P4HA1 serves as a progression marker and potential enzymatic therapeutic target. Furthermore, our studies implicate a role for P4HA1 in prostate cancer progression and invasion *in vitro* and *in vivo*.

Hypoxic conditions in tumor microenvironment are important trigger for metastasis. A recent study by Gilkes et al., [15] showed that hypoxia-induced collagen prolyl hydroxylases promote collagen deposition and induce invasion leading to lymph node and lung metastasis in breast cancer. One of the roles of HIF1α is up-regulating EZH2 expression under hypoxia in breast tumor initiating cells (BTICs) that contribute to cancer progression; HIF inhibitors may potentially be effective in suppressing EZH2 oncogenic function in these cells to prevent cancer recurrence [43]. HIF1α also induces genes that promote angiogenesis, anaerobic metabolism, survival pathways while down-regulating tumor suppressive miR expression [26-28, 44]. In this study, we demonstrated that HIF1α induces *P4HA1*, *EZH2* and *CtBP1* expression. HIF1α, a critical mediator of hypoxia-related metastatic response, was shown earlier [25] and here to induce *P4HA1* expression by directly binding to its promoter. HIF1α therefore up-regulates P4HA1 expression through multiple mechanisms; by directly binding to its promoter and transactivating *P4HA1* promoter or binding to *miR-124* promoter and repressing its expression and thereby indirectly sustaining P4HA1 levels. However, the mechanism of HIF1α-mediated repression of miR-124 gene expression is not completely elucidated. It is possible that the binding of HIF1α to the HRE recruits histone modification complex or other corepressors to *miR-124* promoter and suppresses its transcription. CtBP1 corepressor complex, consisting of histone deacetylases, (HDAC1/2), histone methyl transferases and demethylases is recruited by a large number of transcription factors to mediate sequence-specific transcriptional repression [45]. This explains the enrichment of *miR-124* promoter regions with HIF1α, CtBP1 and EZH2 as well as H3K27me3 mark in the ChIP DNA. Further studies to identify HIF1α interacting partners will elucidate the mechanism of miR-124 down-regulation.

In this study, we show that P4HA1 is regulated by multiple mechanisms in prostate cancer. This study shows copy number gains of *P4HA1* locus as well as the enrichment of HIF1α at *P4HA1* promoter. Our investigation also indicated a role for histone methyltransferase EZH2 in regulating miR-124 expression. EZH2 has been shown to regulate tumor suppressor genes and miRs through histone H3K27 trimethylation marks at their promoters [37, 46]. Additionally, we show here that transcriptional co-repressor CtBP1, which has been shown to repress *LCN2* (Lipocalin-2) and *ARHGDIB* (Rho GDP Dissociation Inhibitor Beta) expression in prostate cancer [16], regulates miR-124 expression.

We identified P4HA1-modulated target genes such as *MMP*s and *FLRT3* by performing gene expression profiling. MMP1 and 2 are known to be involved in prostate cancer progression, invasion and metastasis [25, 39, 47, 48]. We investigated the potential role of P4HA1-induced MMP1 in prostate cancer invasion *in vitro* using either specific siRNA or MMP1 inhibitor (FN-439). Our studies indicated that in prostate epithelial cells over-expressing P4HA1 either MMP1 knockdown or addition of FN-439 significantly reduced MMP1 mediated prostate tumor cell invasion. Our study shows that targeting the enzyme P4HA1 through small molecule inhibitors can achieve results similar to MMP inhibitors in cancer and could be a promising novel therapeutic option. In addition, FLRT3- (a P4HA1-down-regulated gene) rescue experiments demonstrated its role in prostate cancer migration as assessed by the wound healing assay. Our observation is further supported by an earlier study showing that the association of FLRT3 with its cytoplasmic partner, the small GTPase Rnd1 leads to localized down-regulation of C-cadherin to promote cell movements in *Xenopus* [49]. Pharmacological inhibition of P4HA1 using small molecule will potentially allow re-expression and activation of FLRT3 in tumors.

In summary, our study uncovers a complex regulatory axis involving the transcription factor HIF1α, transcriptional repressors CtBP1 and EZH2 that regulate P4HA1 *via* miR-124 (Figure 7F). Overexpression of *P4HA1* in turn attenuates the expression of tumor suppressor FLRT3 and increase expression of genes such as MMPs to trigger invasion and metastasis. This study reveals multiple targets of therapeutic intervention in the P4HA1 pathway in prostate cancer. Thus, as an enzyme, P4HA1 can serve as a promising therapeutic target in prostate cancer.

## METHODS

### Cell Lines

Prostate cancer cell lines DU145, PC3, LnCaP were grown in RPMI 1640 (Life Technologies, CA) with 0.023 IU/ml insulin and 10% FBS (Invitrogen) in 5% CO_2_ cell culture incubator. The HEK-293 (ATCC), PrEC (Lonza, Conshohocken, PA) and RWPE-1(henceforth referred as RWPE; ATCC, Manassas, VA) cells were grown in their respective medium as specified by the suppliers. Lentiviruses were generated by the University of Michigan Vector Core. Prostate cancer cells were infected with lentiviruses expressing P4HA1 shRNA or scramble controls and stable cell lines were generated by selection with 300 μg/ml puromycin (Life Technologies, CA).

### Immunoblot Analyses

Antibodies used in the study are listed in Table S1. All antibodies were employed at dilutions optimized in our laboratory. For immunoblot analysis, 10 μg protein samples were separated on a SDS-PAGE and transferred onto PVDF membrane (GE Healthcare, USA). The membrane was incubated for one hour in blocking buffer (Tris-buffered saline, 0.1% Tween [TBS-T], 5% nonfat dry milk) followed by incubation overnight at 4°C with the primary antibody. After a wash with TBS-T, the blot was incubated with horseradish peroxidase-conjugated secondary antibody and signals were visualized by Pierce enhanced chemiluminescence western blotting substrate as per manufacturer's protocol (Thermo Scientific Inc., USA).

### Gene Expression Analysis

Global gene expression data were generated using RNA isolated from P4HA1 shRNA knockdown DU145 as well as non-target control cells in profiling analysis as well as transcriptome sequencing analysis [8]. Expression profiling was performed using the Agilent Whole Human Genome Oligo Microarray (Agilent, Santa Clara, CA) analysis was performed according to the manufacturer's protocol. Expression values were identified as differential on P4HA1 knockdown if the mean log2(Cy5/Cy3) ratio across cell lines was significantly different from zero as measured by one-sample two sided Student's t tests using a P-value cutoff of 0.05. The list of differentially expressed genes was additionally filtered such that the mean log2 (Cy5/Cy3) ratio exceeded log2(2.5) in absolute value. The resulting list of modulated genes is shown in Figure 6A as a heat map. To measure mRNA expression levels, total RNA was isolated from prostate cell lines and prostate tissue samples using the RNeasy Mini Kit (Qiagen, Valencia, CA). Quantitative real-time polymerase chain reaction (qPCR) was performed as described [16]. All primers were synthesized by Integrated DNA Technologies (Coralville, IA) and PCR reactions were performed in triplicates. Primer sequences used in the present study are listed in Table S2.

### RNA Interference and miRNA Transfection

Small interfering RNA (siRNA) duplexes used to inhibit P4HA1 expression was purchased from Dharmacon, Lafayette, CO. Precursors of respective microRNAs and negative controls were purchased from Ambion (Austin, TX). Transfections were performed with oligofectamine (Life Technologies, NY). Sequence information of all the siRNAs used in this study is listed in Table S3. Short hairpin RNA (shRNA) constructs were generated using pGreen-puro vector for two of the most efficient siRNA duplexes by SBI (System Biosciences, Mountain View, CA). Lentivirus for the stable knockdowns of CtBP1, P4HA1 and HIF1α ShRNA (pGipz HIF-1 (V2LHS: 132152)) was generated by the University of Michigan Vector Core. Lentivirus for the stable knockdowns of EZH2 was generated as described earlier [37]. For miRNA transfection or RNA inference, we plated prostate cancer cell lines DU145, PC3 and RWPE at 1 × 10^5^ cells per well in a 6-well plate and twelve hours later the cells were transfected either with siRNA duplexes or miRNAs using Oligofectamine (Invitrogen, Carlsbad, CA). A second identical transfection was performed 24 hours later. Seventy-two hours after the first transfection, cells were harvested for RNA isolation or immunoblot analysis.

### *In Vitro* Over-expression

P4HA1 cDNA (Origene, MD; Cat# RC223831) was cloned into Gateway expression system (Life Technologies CA). To generate adenoviral and lentiviral constructs, pCR8-P4HA1 (flag-myc tagged) was recombined with pAD/CMV/V5-Dest™ (Life Technologies, CA) or pLenti6/V5-Dest™ (Life Technologies, CA) respectively using LR Clonase II (Life Technologies, CA). Adenoviruses and lentiviruses were generated by the University of Michigan Vector Core. Benign immortalized prostate cells (RWPE) were infected with lentiviruses expressing P4HA1 or lacZ and stable clones were selected with 3.5 μg/ml blasticidin (Santa Cruz Biotechnology, Inc., Dallas, Texas). PrEC and RWPE cells were infected with adenoviruses expressing P4HA1 or lacZ for transient over-expression. EZH2, EZH2ΔSET and CtBP1 adenoviruses were generated as described earlier [37]. To check the secretary MMP1, the RWPE cells were infected with adenoviruses expressing lacZ or P4HA1 and in the presence or absence of MMP1 inhibitor. The culture medium was collected after 48 h incubation and spun down to remove the debris. Then the media is concentrated using Amicon® ultra-4 centrifugal filter tubes (10 Kda molecular weight cut-offs) after following manufacturer's instructions. The concentrate is sonicated and centrifuged 13,000 x g per 15 min. The protein concentration in supernatant was determined using DC™ protein assay (BioRad, USA). These samples were separated on a SDS-PAGE and transferred onto PVDF membrane (GE Healthcare, USA) and followed as described earlier in immunoblotting section.

### Matrigel Invasion Assay

Matrigel invasion assays were performed as described earlier [16, 36, 50]. Various test cells were seeded onto BD BioCoat matrigel matrix (BD, CA) in the upper chamber of a 24-well culture plate. The lower chamber containing respective medium was supplemented with 10% serum as a chemo-attractant. After 48 h, the non-invading cells and matrigel matrix were gently removed with a cotton swab. Invasive cells located on the lower side of the chamber were stained with 0.2 % crystal violet in methanol, air-dried and photographed using an inverted microscope (4X). Invasion was quantified either by colorimetric assay or cell counting. For colorimetric assays, the inserts were treated with 150 μl of 10% acetic acid and the absorbance measured at 560 nm.

### Chromatin Immunoprecipitation (ChIP) Assays

We screened the human *P4HA1*, *VEGFA*, *GLUT-1*, *miR-124-1*,*-2* and *-3* promoters with Genomatix MatInspector and detected several HIF1α-binding sites. Using UCSC (University of California Santa Cruz) genome browser, we detected H3K27me3 marks and EZH2 binding sites on *miR-124-1*,*-2* and *-3* promoters [51]. A few microRNA promoters have been identified experimentally; a 6-kb region (5 kb upstream and 1 kb downstream region of the 5′ end of the annotated microRNA) was designated as a putative promoter sequence [29]. For CtBP1, EZH2 and H3K27me3 ChIP assay, we searched for possible binding sites in the *miR-124-1*,*-2* and *-3* promoter regions within -4 to 0 kb of the transcriptional start site (TSS) [52, 53] and designed primers accordingly. ChIP assays were carried out with respective antibodies (Table S1) using the EZ-Magna ChIP kit (Millipore, Billerica, MA) as described [16]. Briefly, 5 × 10^6^ cells were cross-linked by addition of formaldehyde to a final concentration of 1 % for 10 minutes. Cross-linking was terminated with glycine (final concentration of 0.125 M) followed by cell lysis and sonication, resulting in an average fragment size of 500 bp. Antibody incubations were carried out over-night at 4 °C. Reversal of cross-linking was carried out at 65 °C for 3 hours followed by DNA isolation. The purified DNA was analyzed by qPCR to determine fold enrichment relative to IgG. The primer sequences for the promoters analyzed are provided in Table S4 and S5 (for HIF1α-ChIP), S6 (for CtBP1/EZH2-ChIP) and S7 (for trimethyl-H3K27-ChIP).

### Prostate Tumor Xenograft Model

All procedures involving mice were approved by the University Committee on Use and Care of Animals (UCUCA) at the University of Michigan and conform to all regulatory standards. To evaluate the role of P4HA1 in tumor formation *in vivo*, we propagated stable P4HA1 knockdown PC3 pools using two-independent shRNAs and vector control cells, and inoculated 1 × 10^6^ cells subcutaneously into the dorsal flank of 5-week-old male nude Athymic nude mice (n = 8 for each group; Harlan Laboratories, MI). Tumor size was measured biweekly, and tumor volumes were calculated using the formula (π/6) (L × W^2^), where L = length and W = width of the tumor.

## SUPPLEMENTARY MATERIAL FIGURES AND TABLES



## References

[1] Siegel R, Ma J, Zou Z, Jemal A (2014). Cancer statistics, 2014. CA: a cancer journal for clinicians.

[2] Alizadeh AA, Eisen MB, Davis RE, Ma C, Lossos IS, Rosenwald A, Boldrick JC, Sabet H, Tran T, Yu X, Powell JI, Yang L, Marti GE, Moore T, Hudson J, Lu L (2000). Distinct types of diffuse large B-cell lymphoma identified by gene expression profiling. Nature.

[3] Dhanasekaran SM, Barrette TR, Ghosh D, Shah R, Varambally S, Kurachi K, Pienta KJ, Rubin MA, Chinnaiyan AM (2001). Delineation of prognostic biomarkers in prostate cancer. Nature.

[4] van de Vijver MJ, He YD, van't Veer LJ, Dai H, Hart AA, Voskuil DW, Schreiber GJ, Peterse JL, Roberts C, Marton MJ, Parrish M, Atsma D, Witteveen A, Glas A, Delahaye L, van der Velde T (2002). A gene-expression signature as a predictor of survival in breast cancer. The New England journal of medicine.

[5] van 't Veer LJ, Dai H, van de Vijver MJ, He YD, Hart AA, Mao M, Peterse HL, van der Kooy K, Marton MJ, Witteveen AT, Schreiber GJ, Kerkhoven RM, Roberts C, Linsley PS, Bernards R, Friend SH (2002). Gene expression profiling predicts clinical outcome of breast cancer. Nature.

[6] Armstrong SA, Staunton JE, Silverman LB, Pieters R, den Boer ML, Minden MD, Sallan SE, Lander ES, Golub TR, Korsmeyer SJ (2002). MLL translocations specify a distinct gene expression profile that distinguishes a unique leukemia. Nature genetics.

[7] Varambally S, Dhanasekaran SM, Zhou M, Barrette TR, Kumar-Sinha C, Sanda MG, Ghosh D, Pienta KJ, Sewalt RG, Otte AP, Rubin MA, Chinnaiyan AM (2002). The polycomb group protein EZH2 is involved in progression of prostate cancer. Nature.

[8] Krek A, Grun D, Poy MN, Wolf R, Rosenberg L, Epstein EJ, MacMenamin P, da Piedade I, Gunsalus KC, Stoffel M, Rajewsky N (2005). Combinatorial microRNA target predictions. Nat Genet.

[9] Ferreira PG, Jares P, Rico D, Gomez-Lopez G, Martinez-Trillos A, Villamor N, Ecker S, Gonzalez-Perez A, Knowles DG, Monlong J, Johnson R, Quesada V, Djebali S, Papasaikas P, Lopez-Guerra M, Colomer D (2014). Transcriptome characterization by RNA sequencing identifies a major molecular and clinical subdivision in chronic lymphocytic leukemia. Genome research.

[10] Palanisamy N, Ateeq B, Kalyana-Sundaram S, Pflueger D, Ramnarayanan K, Shankar S, Han B, Cao Q, Cao X, Suleman K, Kumar-Sinha C, Dhanasekaran SM, Chen YB, Esgueva R, Banerjee S, LaFargue CJ (2010). Rearrangements of the RAF kinase pathway in prostate cancer, gastric cancer and melanoma. Nature medicine.

[11] Myllyharju J (2003). Prolyl 4-hydroxylases, the key enzymes of collagen biosynthesis. Matrix Biol.

[12] Myllyharju J, Kivirikko KI (2004). Collagens, modifying enzymes and their mutations in humans, flies and worms. Trends Genet.

[13] Hanahan D, Weinberg RA (2011). Hallmarks of cancer: the next generation. Cell.

[14] Gilkes DM, Bajpai S, Chaturvedi P, Wirtz D, Semenza GL (2013). Hypoxia-inducible Factor 1 (HIF-1) Promotes Extracellular Matrix Remodeling under Hypoxic Conditions by Inducing P4HA1, P4HA2, and PLOD2 Expression in Fibroblasts. J Biol Chem.

[15] Gilkes DM, Chaturvedi P, Bajpai S, Wong CC, Wei H, Pitcairn S, Hubbi ME, Wirtz D, Semenza GL (2013). Collagen prolyl hydroxylases are essential for breast cancer metastasis. Cancer Res.

[16] Wang R, Asangani IA, Chakravarthi BV, Ateeq B, Lonigro RJ, Cao Q, Mani RS, Camacho DF, McGregor N, Schumann TE, Jing X, Menawat R, Tomlins SA, Zheng H, Otte AP, Mehra R (2012). Role of transcriptional corepressor CtBP1 in prostate cancer progression. Neoplasia.

[17] Grasso CS, Wu YM, Robinson DR, Cao X, Dhanasekaran SM, Khan AP, Quist MJ, Jing X, Lonigro RJ, Brenner JC, Asangani IA, Ateeq B, Chun SY, Siddiqui J, Sam L, Anstett M (2012). The mutational landscape of lethal castration-resistant prostate cancer. Nature.

[18] Prensner JR, Iyer MK, Balbin OA, Dhanasekaran SM, Cao Q, Brenner JC, Laxman B, Asangani IA, Grasso CS, Kominsky HD, Cao X, Jing X, Wang X, Siddiqui J, Wei JT, Robinson D (2011). Transcriptome sequencing across a prostate cancer cohort identifies PCAT-1, an unannotated lincRNA implicated in disease progression. Nature biotechnology.

[19] Kalyana-Sundaram S, Kumar-Sinha C, Shankar S, Robinson DR, Wu YM, Cao X, Asangani IA, Kothari V, Prensner JR, Lonigro RJ, Iyer MK, Barrette T, Shanmugam A, Dhanasekaran SM, Palanisamy N, Chinnaiyan AM (2012). Expressed pseudogenes in the transcriptional landscape of human cancers. Cell.

[20] Vanaja DK, Cheville JC, Iturria SJ, Young CY (2003). Transcriptional silencing of zinc finger protein 185 identified by expression profiling is associated with prostate cancer progression. Cancer Res.

[21] Yu YP, Landsittel D, Jing L, Nelson J, Ren B, Liu L, McDonald C, Thomas R, Dhir R, Finkelstein S, Michalopoulos G, Becich M, Luo JH (2004). Gene expression alterations in prostate cancer predicting tumor aggression and preceding development of malignancy. Journal of clinical oncology : official journal of the American Society of Clinical Oncology.

[22] Lewis BP, Shih IH, Jones-Rhoades MW, Bartel DP, Burge CB (2003). Prediction of mammalian microRNA targets. Cell.

[23] John B, Enright AJ, Aravin A, Tuschl T, Sander C, Marks DS (2004). Human MicroRNA targets. PLoS biology.

[24] Lewis BP, Burge CB, Bartel DP (2005). Conserved seed pairing, often flanked by adenosines, indicates that thousands of human genes are microRNA targets. Cell.

[25] Bentovim L, Amarilio R, Zelzer E (2012). HIF1alpha is a central regulator of collagen hydroxylation and secretion under hypoxia during bone development. Development.

[26] Cao P, Deng Z, Wan M, Huang W, Cramer SD, Xu J, Lei M, Sui G (2010). MicroRNA-101 negatively regulates Ezh2 and its expression is modulated by androgen receptor and HIF-1alpha/HIF-1beta. Molecular cancer.

[27] Du R, Sun W, Xia L, Zhao A, Yu Y, Zhao L, Wang H, Huang C, Sun S (2012). Hypoxia-induced down-regulation of microRNA-34a promotes EMT by targeting the Notch signaling pathway in tubular epithelial cells. PLoS One.

[28] He M, Wang QY, Yin QQ, Tang J, Lu Y, Zhou CX, Duan CW, Hong DL, Tanaka T, Chen GQ, Zhao Q (2013). HIF-1alpha downregulates miR-17/20a directly targeting p21 and STAT3: a role in myeloid leukemic cell differentiation. Cell death and differentiation.

[29] Kulshreshtha R, Ferracin M, Wojcik SE, Garzon R, Alder H, Agosto-Perez FJ, Davuluri R, Liu CG, Croce CM, Negrini M, Calin GA, Ivan M (2007). A microRNA signature of hypoxia. Molecular and cellular biology.

[30] Gray MJ, Zhang J, Ellis LM, Semenza GL, Evans DB, Watowich SS, Gallick GE (2005). HIF-1alpha, STAT3, CBP/p300 and Ref-1/APE are components of a transcriptional complex that regulates Src-dependent hypoxia-induced expression of VEGF in pancreatic and prostate carcinomas. Oncogene.

[31] Hu CJ, Iyer S, Sataur A, Covello KL, Chodosh LA, Simon MC (2006). Differential regulation of the transcriptional activities of hypoxia-inducible factor 1 alpha (HIF-1alpha) and HIF-2alpha in stem cells. Molecular and cellular biology.

[32] Furuta M, Kozaki KI, Tanaka S, Arii S, Imoto I, Inazawa J (2010). miR-124 and miR-203 are epigenetically silenced tumor-suppressive microRNAs in hepatocellular carcinoma. Carcinogenesis.

[33] Gebauer K, Peters I, Dubrowinskaja N, Hennenlotter J, Abbas M, Scherer R, Tezval H, Merseburger AS, Stenzl A, Kuczyk MA, Serth J (2013). Hsa-mir-124-3 CpG island methylation is associated with advanced tumours and disease recurrence of patients with clear cell renal cell carcinoma. British journal of cancer.

[34] Sato F, Tsuchiya S, Meltzer SJ, Shimizu K (2011). MicroRNAs and epigenetics. The FEBS journal.

[35] Wang P, Chen L, Zhang J, Chen H, Fan J, Wang K, Luo J, Chen Z, Meng Z, Liu L (2014). Methylation-mediated silencing of the miR-124 genes facilitates pancreatic cancer progression and metastasis by targeting Rac1. Oncogene.

[36] Asangani IA, Ateeq B, Cao Q, Dodson L, Pandhi M, Kunju LP, Mehra R, Lonigro RJ, Siddiqui J, Palanisamy N, Wu YM, Cao X, Kim JH, Zhao M, Qin ZS, Iyer MK (2013). Characterization of the EZH2-MMSET histone methyltransferase regulatory axis in cancer. Molecular cell.

[37] Cao Q, Mani RS, Ateeq B, Dhanasekaran SM, Asangani IA, Prensner JR, Kim JH, Brenner JC, Jing X, Cao X, Wang R, Li Y, Dahiya A, Wang L, Pandhi M, Lonigro RJ (2011). Coordinated regulation of polycomb group complexes through microRNAs in cancer. Cancer Cell.

[38] Karaulanov E, Bottcher RT, Stannek P, Wu W, Rau M, Ogata S, Cho KW, Niehrs C (2009). Unc5B interacts with FLRT3 and Rnd1 to modulate cell adhesion in Xenopus embryos. PLoS One.

[39] Pulukuri SM, Rao JS (2008). Matrix metalloproteinase-1 promotes prostate tumor growth and metastasis. International journal of oncology.

[40] Odake S, Morita Y, Morikawa T, Yoshida N, Hori H, Nagai Y (1994). Inhibition of matrix metalloproteinases by peptidyl hydroxamic acids. Biochemical and biophysical research communications.

[41] Boire A, Covic L, Agarwal A, Jacques S, Sherifi S, Kuliopulos A (2005). PAR1 is a matrix metalloprotease-1 receptor that promotes invasion and tumorigenesis of breast cancer cells. Cell.

[42] Rhodes DR, Kalyana-Sundaram S, Mahavisno V, Varambally R, Yu J, Briggs BB, Barrette TR, Anstet MJ, Kincead-Beal C, Kulkarni P, Varambally S, Ghosh D, Chinnaiyan AM (2007). Oncomine 3. 0: genes, pathways, and networks in a collection of 18,000 cancer gene expression profiles. Neoplasia.

[43] Chang CJ, Yang JY, Xia W, Chen CT, Xie X, Chao CH, Woodward WA, Hsu JM, Hortobagyi GN, Hung MC (2011). EZH2 promotes expansion of breast tumor initiating cells through activation of RAF1-beta-catenin signaling. Cancer Cell.

[44] Koh MY, Spivak-Kroizman TR, Powis G (2010). HIF-1alpha and cancer therapy. Recent results in cancer research Fortschritte der Krebsforschung Progres dans les recherches sur le cancer.

[45] Kuppuswamy M, Vijayalingam S, Zhao LJ, Zhou Y, Subramanian T, Ryerse J, Chinnadurai G (2008). Role of the PLDLS-binding cleft region of CtBP1 in recruitment of core and auxiliary components of the corepressor complex. Molecular and cellular biology.

[46] Varambally S, Cao Q, Mani RS, Shankar S, Wang X, Ateeq B, Laxman B, Cao X, Jing X, Ramnarayanan K, Brenner JC, Yu J, Kim JH, Han B, Tan P, Kumar-Sinha C (2008). Genomic loss of microRNA-101 leads to overexpression of histone methyltransferase EZH2 in cancer. Science.

[47] Dong Z, Bonfil RD, Chinni S, Deng X, Trindade Filho JC, Bernardo M, Vaishampayan U, Che M, Sloane BF, Sheng S, Fridman R, Cher ML (2005). Matrix metalloproteinase activity and osteoclasts in experimental prostate cancer bone metastasis tissue. The American journal of pathology.

[48] Pratap J, Javed A, Languino LR, van Wijnen AJ, Stein JL, Stein GS, Lian JB (2005). The Runx2 osteogenic transcription factor regulates matrix metalloproteinase 9 in bone metastatic cancer cells and controls cell invasion. Molecular and cellular biology.

[49] Chen X, Koh E, Yoder M, Gumbiner BM (2009). A protocadherin-cadherin-FLRT3 complex controls cell adhesion and morphogenesis. PLoS One.

[50] Tomlins SA, Laxman B, Varambally S, Cao X, Yu J, Helgeson BE, Cao Q, Prensner JR, Rubin MA, Shah RB, Mehra R, Chinnaiyan AM (2008). Role of the TMPRSS2-ERG gene fusion in prostate cancer. Neoplasia.

[51] Kent WJ, Sugnet CW, Furey TS, Roskin KM, Pringle TH, Zahler AM, Haussler D (2002). The human genome browser at UCSC. Genome research.

[52] Deng Y, Liu J, Han G, Lu SL, Wang SY, Malkoski S, Tan AC, Deng C, Wang XJ, Zhang Q (2010). Redox-dependent Brca1 transcriptional regulation by an NADH-sensor CtBP1. Oncogene.

[53] Cakouros D, Isenmann S, Cooper L, Zannettino A, Anderson P, Glackin C, Gronthos S (2012). Twist-1 induces Ezh2 recruitment regulating histone methylation along the Ink4A/Arf locus in mesenchymal stem cells. Molecular and cellular biology.

