# Supplementary material for: The miR-124-Prolyl Hydroxylase P4HA1-MMP1 axis plays a critical role in prostate cancer progression
Source: Oncotarget. 2014 Jul 12;5(16):6654–69. doi: 10.18632/oncotarget.2208 (PMC4196154; doi:10.18632/oncotarget.2208)
Supplement: Supplementary file 1 [file oncotarget-05-6654-s001.docx]

### SUPPLEMENTARY MATERIAL

**The miR-124-Prolyl Hydroxylase P4HA1-MMP1 axis plays a critical role in prostate cancer progression**

Balabhadrapatruni V. S. K. Chakravarthi^1,2^, Satya S. Pathi^1,2,7^, Moloy T. Goswami^1,2,7^, Marcin Cieślik^1,2^, Heng Zheng^1^, Sivakumar Nallasivam^1^, Subramanyeswara R. Arekapudi^1,6^, Xiaojun Jing^1,2^, Javed Siddiqui^1,2^, Jyoti Athanikar^1,2^, Shannon L. Carskadon^1,2^, Robert J. Lonigro^1,2^, Lakshmi P. Kunju^1,2^, Arul M. Chinnaiyan^1,2,3,4,5^, Nallasivam Palanisamy^1,2,5^, Sooryanarayana Varambally^1,2,5^

^1^ Michigan Center for Translational Pathology

^2^ Department of Pathology, University of Michigan

^3^ Department of Urology, University of Michigan

^4^ Howard Hughes Medical Institute, University of Michigan Medical School

^5^ Comprehensive Cancer Center, University of Michigan Medical School, Ann Arbor, MI 48109, USA

^6^Present Address: Department of Hematology and Oncology, Providence Hospital and Medical Center, Southfield, MI 48075, USA

^7^Contributed equally

**Corresponding Author**

Sooryanarayana Varambally, Ph.D., Michigan Center for Translational Pathology, Department of Pathology, University of Michigan Medical School, 2900 Huron Parkway, Traverwood IV.

Ann Arbor, Michigan 48109-0602, Phone: (734) 232-0812, Fax: (734) 615-4055

Email: [soory@med.umich.edu](mailto:soory@med.umich.edu)

**INVENTORY**

**SUPLLEMENTAL DATA**

Supplementary Fig. S1, related to Figure 1.

Supplementary Fig. S2, related to Figure 2.

Supplementary Fig. S3, related to Figure 2.

Supplementary Fig. S4, related to Figure 3.

Supplementary Fig. S5, related to Figure 4.

Supplementary Fig. S6, related to Figure 4.

Supplementary Fig. S7, related to Figure 4.

Supplementary Fig. S8, related to Figure 5.

Supplementary Fig. S9, related to Figure 5.

Supplementary Fig. S10, related to Figure 5.

Supplementary Fig. S11, related to Figure 6.

Supplementary Fig. S12, related to Figure 6.

Supplementary Fig. S13, related to Figure 6.

Supplementary Fig. S14, related to Figures 6.

Table S1, related to Extended Experimental Procedures.

Table S2, related to Extended Experimental Procedures.

Table S3, related to Extended Experimental Procedures.

Table S4, related to Extended Experimental Procedures.

Table S5, related to Extended Experimental Procedures.

Table S6, related to Extended Experimental Procedures.

Table S7, related to Extended Experimental Procedures.

**EXTENDED EXPERIMENTAL PROCEDURES**

**SUPPLEMENTAL REFERENCES**

###
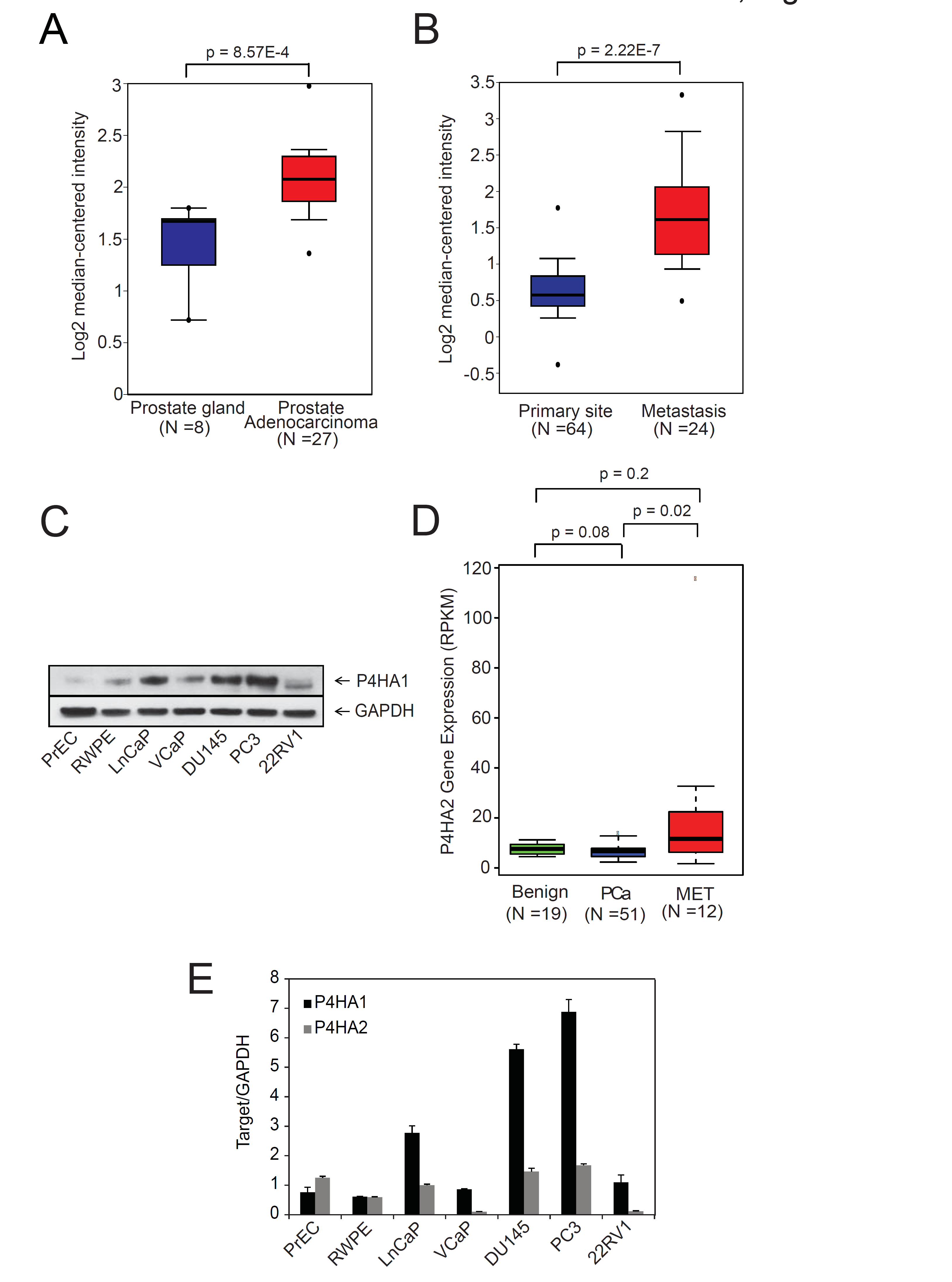


**Supplementary Figure S1,** **Related to Figure 1: P4HA1 expression, but not P4HA2, is high in metastatic prostate cancer cell lines.**

**(A, B)** Box plots represent *P4HA1* expression level in **(A)** prostate adenocarcinoma and, **(B)** metastatic samples. The data was retrieved from publicly available microarray datasets of prosate cancer with log2 median intensity for Oncomine array datasets. **(C)** P4HA1 protein levels in various cell lines by immunoblot analysis. **(D)** *P4HA2* expression levels in benign, prostate carcinoma and metastatic prostate cancer (MET) samples. **(E)** *P4HA1* and *P4HA2* expression in prostate cancer cell lines. All bar graphs are shown with ± SEM.

###
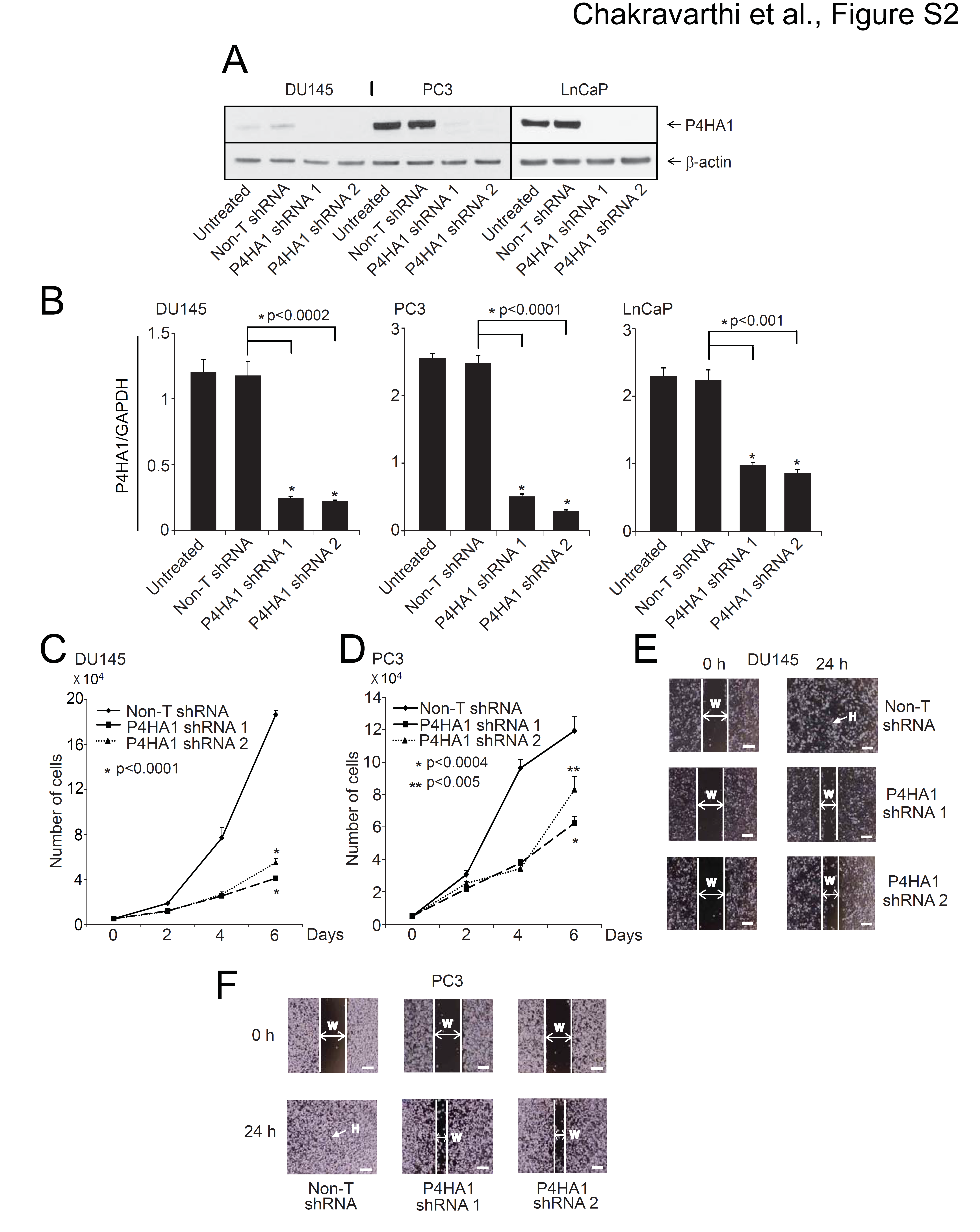


**Supplementary Figure S2, Related to Figure 2:** **P4HA1 stable knockdown markedly decreases cell proliferation and motility in prostate cancer cells.**

**(A)** Immunoblot analysis showing P4HA1 protein expression in DU145, PC3 and LnCaP cells transduced with two independent P4HA1 shRNA duplexes or control non-targeting shRNA. **(B)** qPCR analysis of *P4HA1* in cells from **(A)**. **(C, D)** Stable knockdown of P4HA1 reduces prostate cancer cell proliferation. Cell proliferation was measured using cells transduced with P4HA1 shRNA duplex or control non-targeting shRNA in DU145 and PC3 cells. **(E, F)** Wound healing assay, an artificial wound was created using a 0.2 ml pipette tip on a confluent monolayer of cells. Images were taken at 0 and 24 h after scratching. The white lines show the margin of scratched area in which double headed arrow indicates scratch width (W) and white arrow indicates complete healing (H) of scratch wound. These images show that the migration was slower in P4HA1 stable knockdown prostate cancer cells compared to the cells expressing non-targeting shRNA. This assay was performed in three independent experiments. All bar graphs are shown with ± SEM.

###
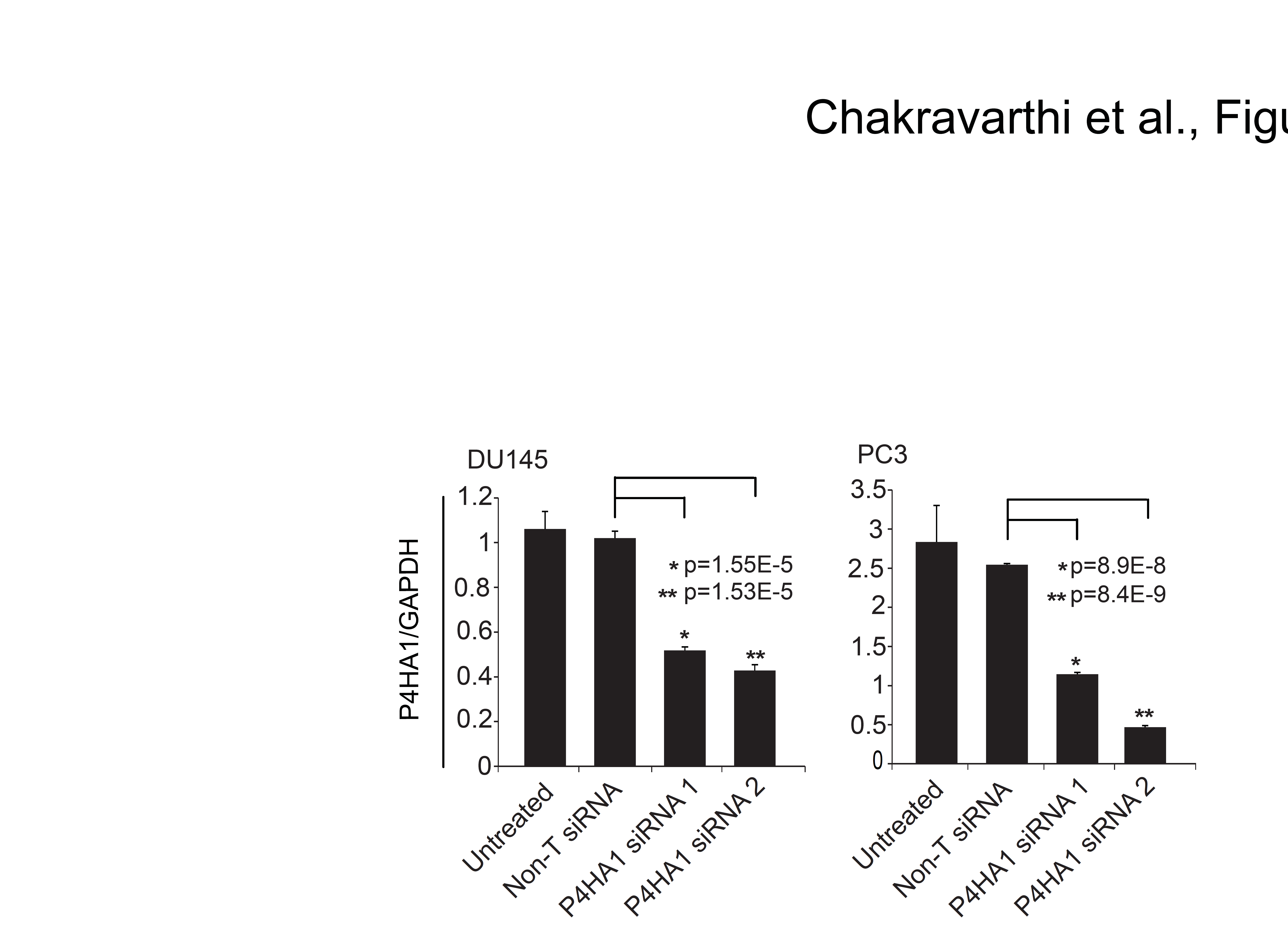


**Supplementary Figure S3, Related to Figure 2: qPCR analysis of *P4HA1* in knockdown cells.**

qPCR was performed in cells treated either with P4HA1 siRNA duplex or non-targeting siRNA control using DU145 and PC3 cells. GAPDH is used as an internal control. All bar graphs are shown with ± SEM.

###
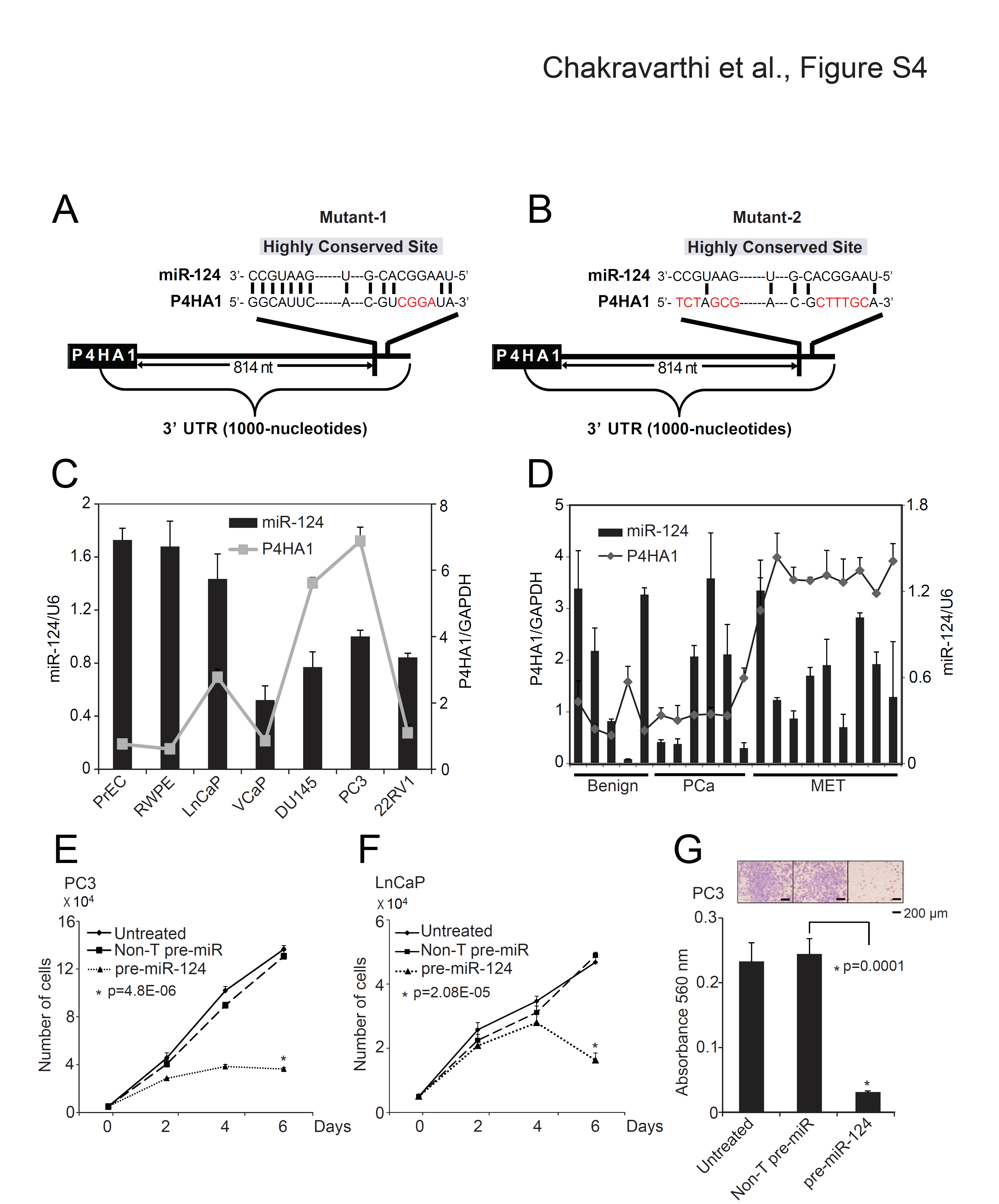


### Supplementary Figure S4, Related to Figure 3: miR-124 regulates cell proliferation and invasion by binding to P4HA1-3’-UTR.

**(A)** miR-124 binding sites in P4HA1 3’-UTR. Schematic representation of mutant-1 and **(B)** mutant-2 P4HA1 3’-UTRs. Red nucleotides represent mutations that are predicted to disrupt potential miR-124 binding. **(C)** miR-124 and *P4HA1* expression levels are inversely correlated. miR-124 and *P4HA1* expression levels were assessed both in prostate cancer cell lines and **(D)** tissue specimens. **(E, F)** miR-124 reduces cell proliferation in PC3 and LnCaP cells. Cell proliferation was measured in PC3 and LnCaP cells overexpressing miR-124. **(G)** Pre-miR-124-treated PC3 cells showed reduced invasion in Boyden chamber matrigel invasion assay. Invaded cells were stained with crystal violet and measured the absorbance at 560 nm. All bar graphs are shown with ± SEM.

###
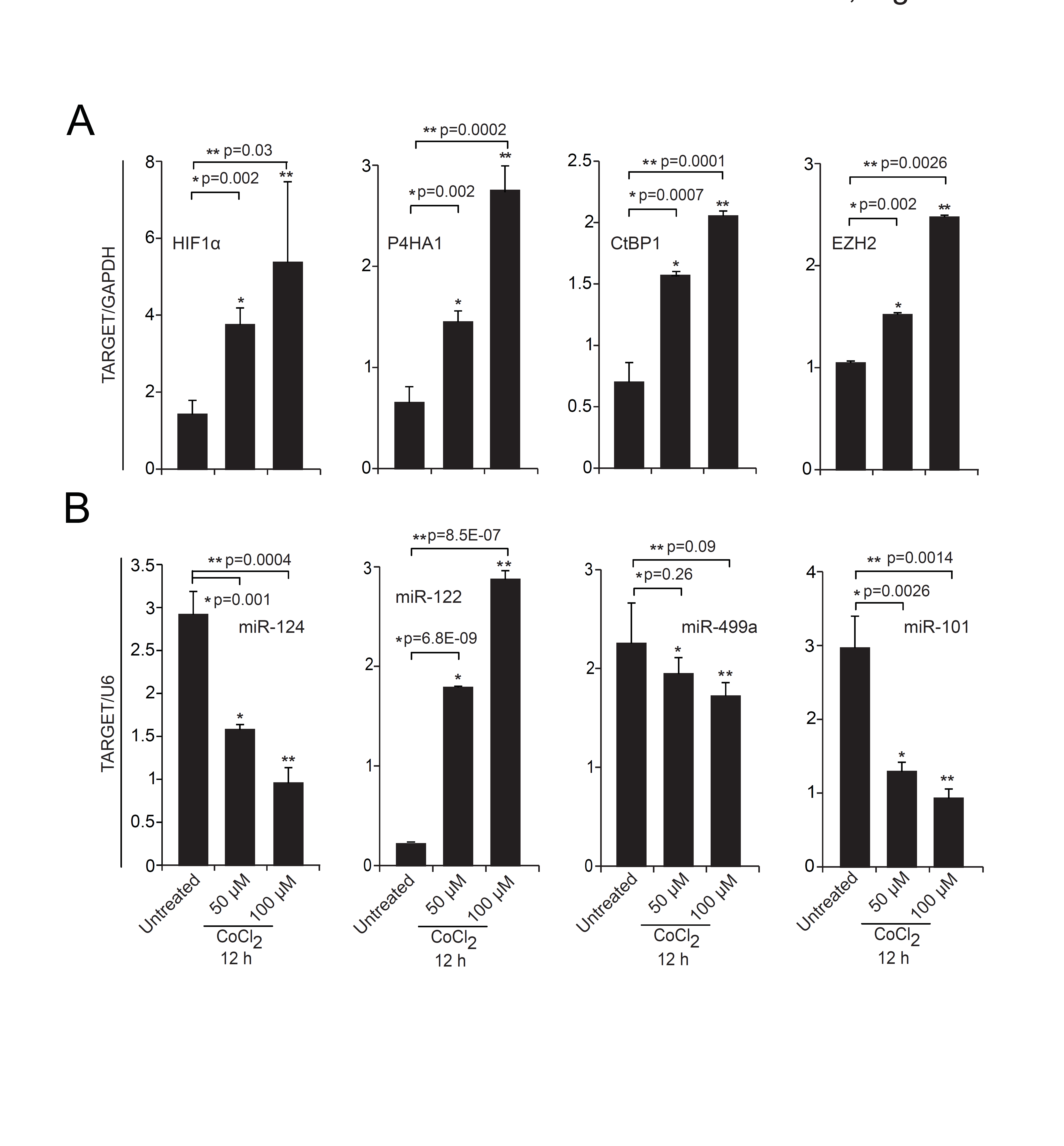


**Supplementary Figure S5, Related to Figure 4: miR-124 is down-regulated under hypoxia-mimicking conditions.**

**(A)** qPCR analysis of *HIF1α*, *P4HA1*, *CtBP1* and *EZH2*, and **(B)** miR-124, 122, 499a and 101 in RWPE cells in the presence of hypoxia-mimetic agent CoCl_2_. All bar graphs are shown with ± SEM.

###

###
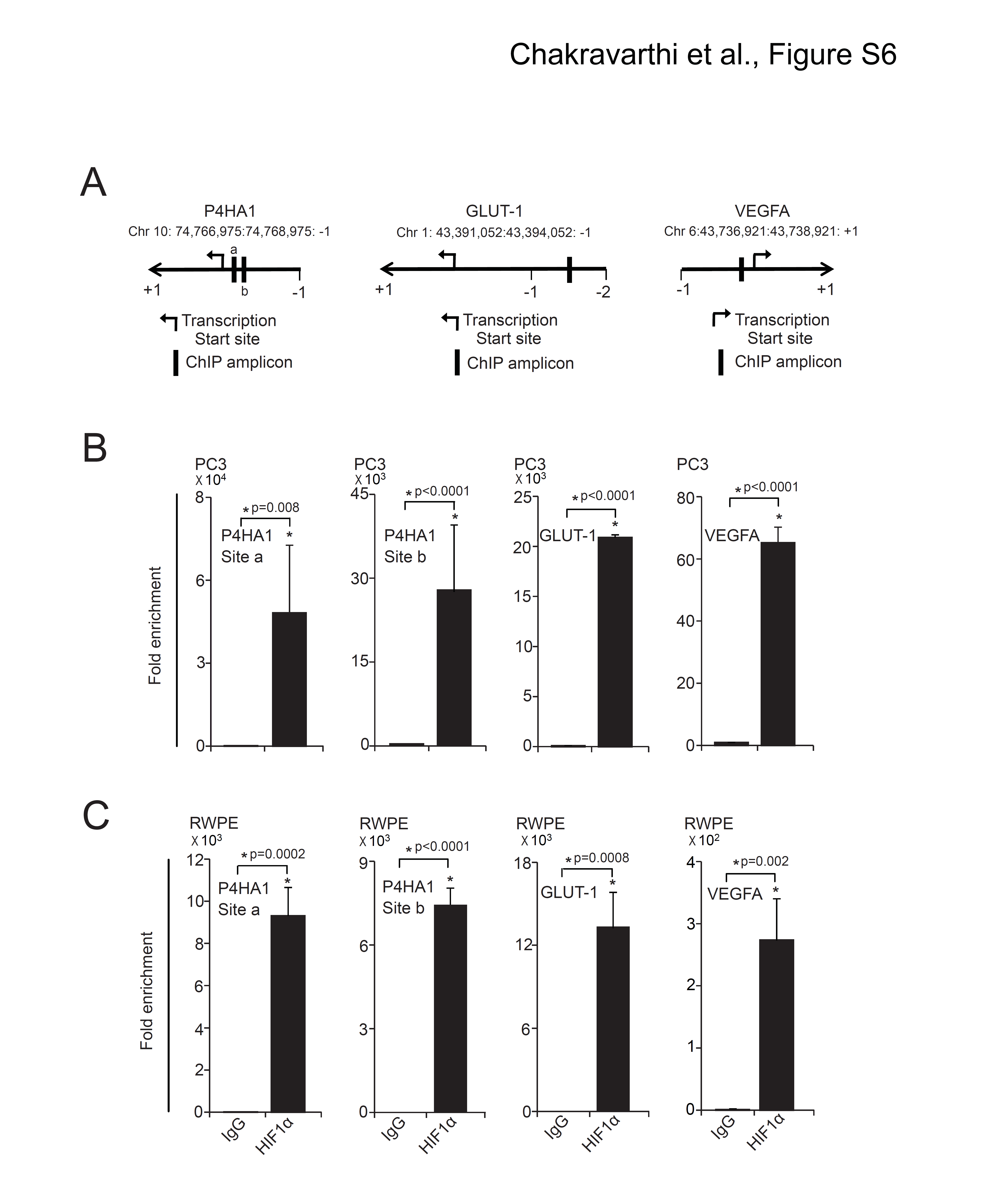


### Supplementary Figure S6, Related to Figure 4: HIF1α occupies *P4HA1*, *GLUT-1* and *VEGFA* promoters.

Schematic representation of the **(A)** *P4HA1*, *GLUT-1* and *VEGFA* genomic region on chromosome 10, 1 and 6 respectively, showing transcription start site and amplicon positions. Conventional Chromatin immunoprecipitation (ChIP)-PCR analysis of HIF1α occupancy on *P4HA1*, *GLUT-1* and *VEGFA* promoters in **(B)** PC3 and **(C)** RWPE cells following induction with 100 µM CoCl_2_ for 12 h. ChIP was performed using antibodies against HIF1α and a control IgG. Error bars: n = 3. All bar graphs are shown with ± SEM.

###
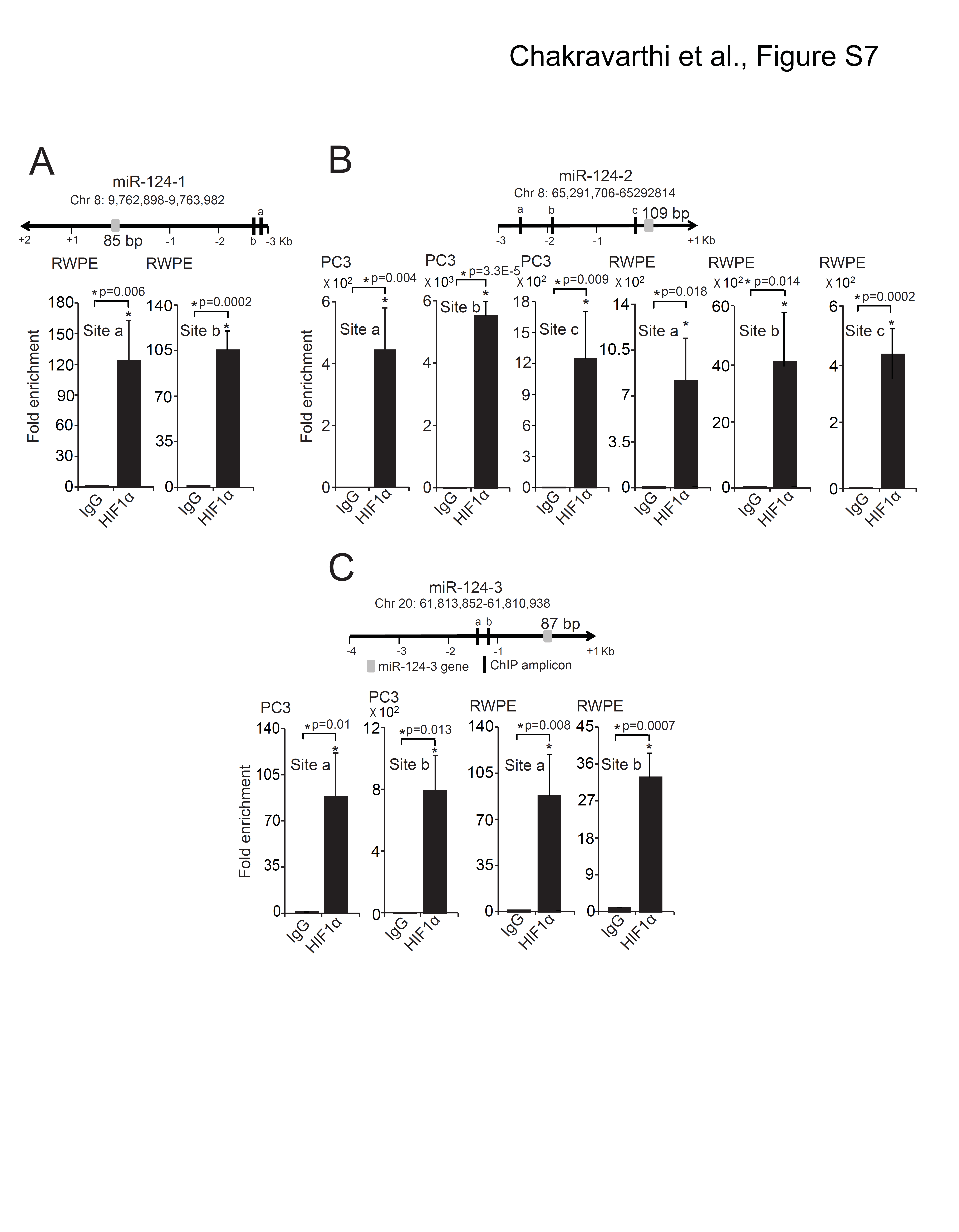


**Supplementary Figure S7, Related to Figure 4: HIF1α occupies *miR-124* promoter.**

**(A, B, C)** Conventional Chromatin immunoprecipitation (ChIP)-PCR analysis of HIF1α occupancy on *miR-124-1*, *-2* and *-3* promoter sites in PC3 and RWPE cells following induction with 100 µM CoCl_2_ for 12 h. ChIP was performed using antibodies against HIF1α and a control IgG. Inset: Schematic representation of the *miR-124-1*, *miR-124-2* and *miR-124-3* genomic regions on chromosome 8 and 20 respectively, showing gene and amplicon positions. Error bars: n = 3. All bar graphs are shown with ± SEM.

###
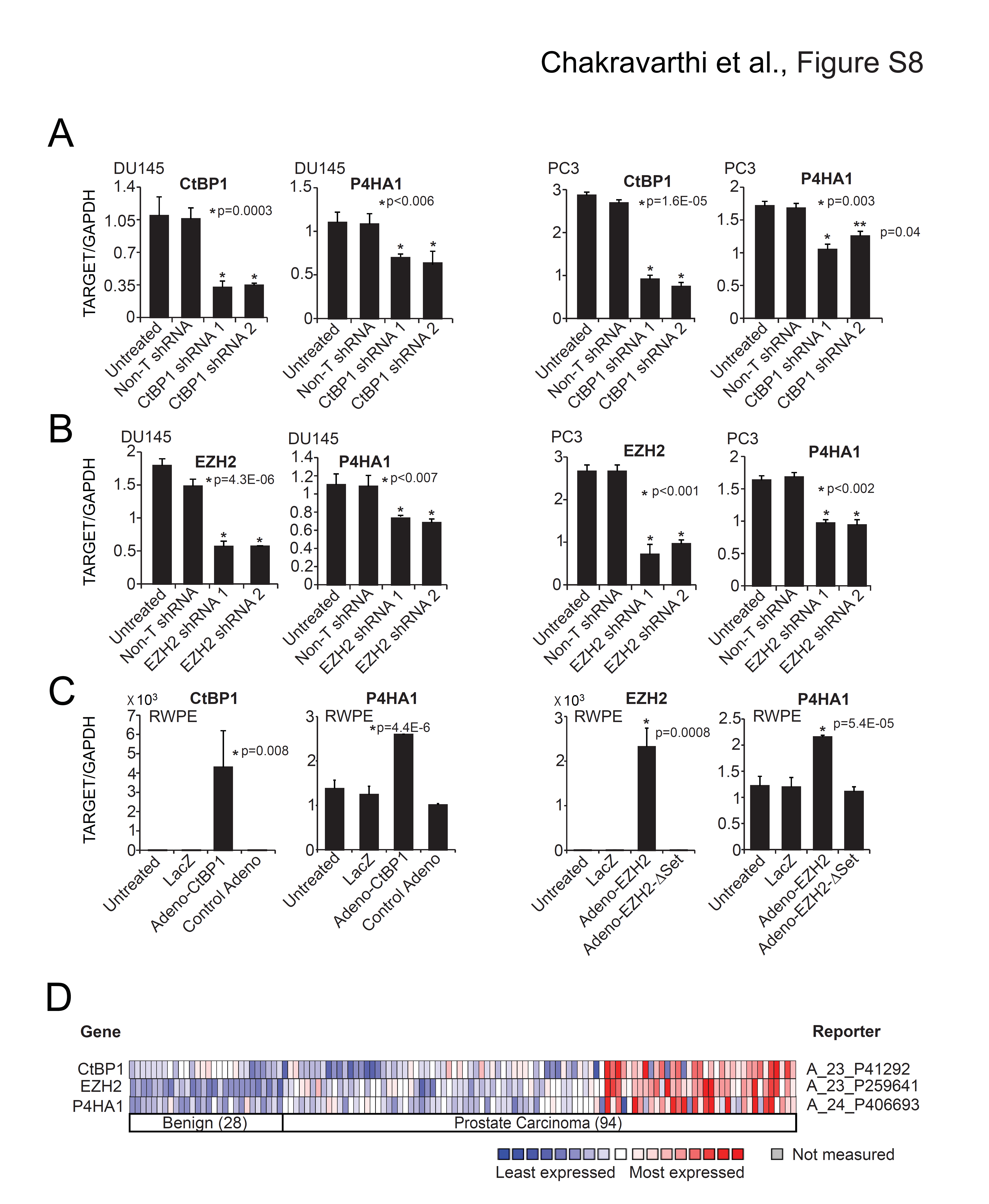


**Supplementary Figure S8, Related to Figure 5: CtBP1 and EZH2 regulate P4HA1 expression.**

**(A)** qPCR analysis of *CtBP1* and *P4HA1* in CtBP1 stable knockdown DU145 and PC3 cells and **(B)** *EZH2* and *P4HA1* in EZH2 stable knockdown DU145 and PC3 cells. **(C)** qPCR analysis of *CtBP1*, *EZH2* and *P4HA1* in benign prostate cell line RWPE cells following infection with control, lacZ adenovirus or CtBP1, EZH2 or EZH2ΔSET mutant adenovirus for 48 h. **(D)** Expression of *P4HA1*, *CtBP1* and *EZH2* are positively correlated. Heat map of *P4HA1*, *CtBP1* and *EZH2* expression levels across prostate benign, carcinoma and metastatic samples. The data was retrieved from Oncomine (Grasso et al., 2012). Blue and red color bars signify the lowest and highest levels of expression respectively (log2 median-centered ratio). All bar graphs are shown with ± SEM.

###
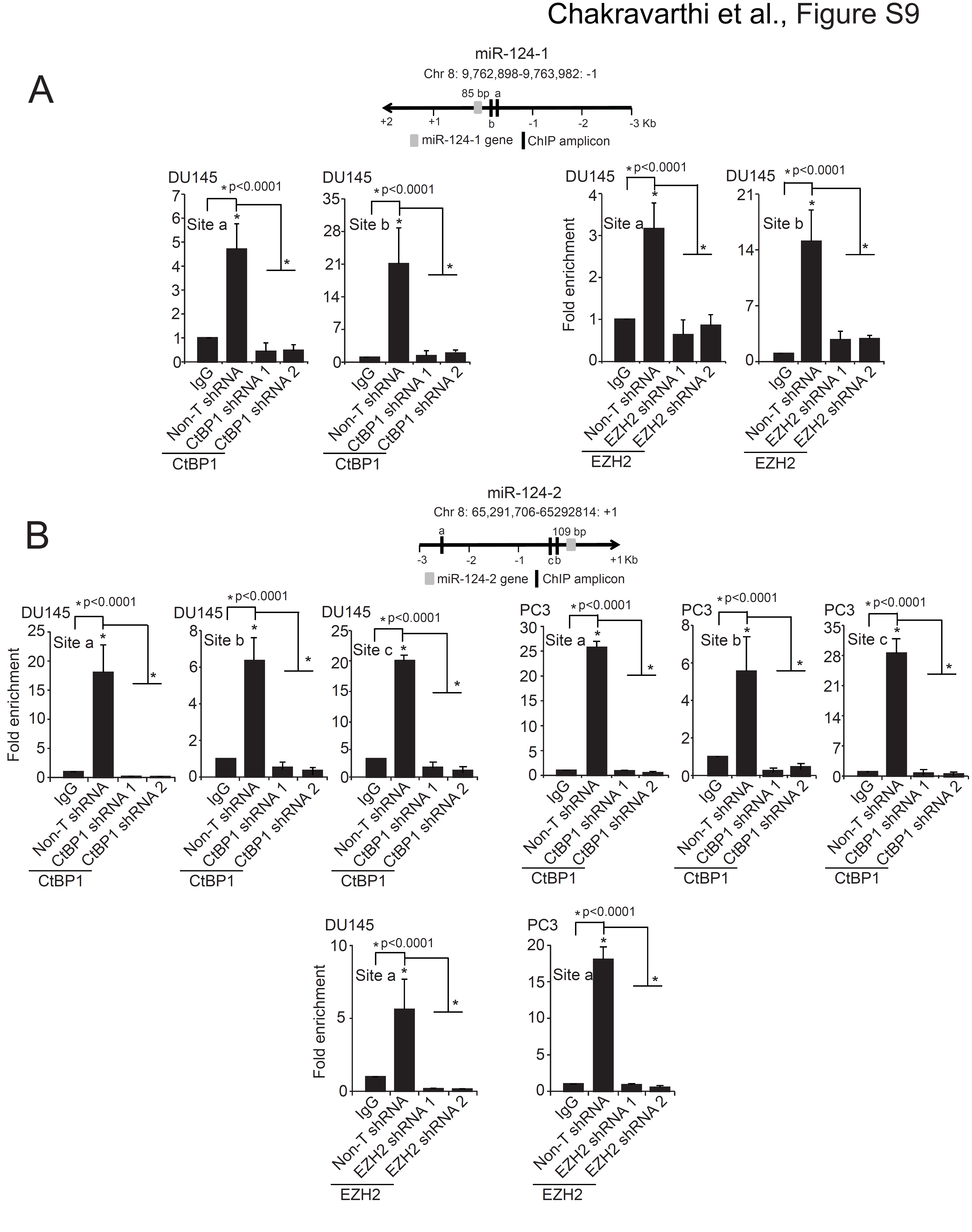


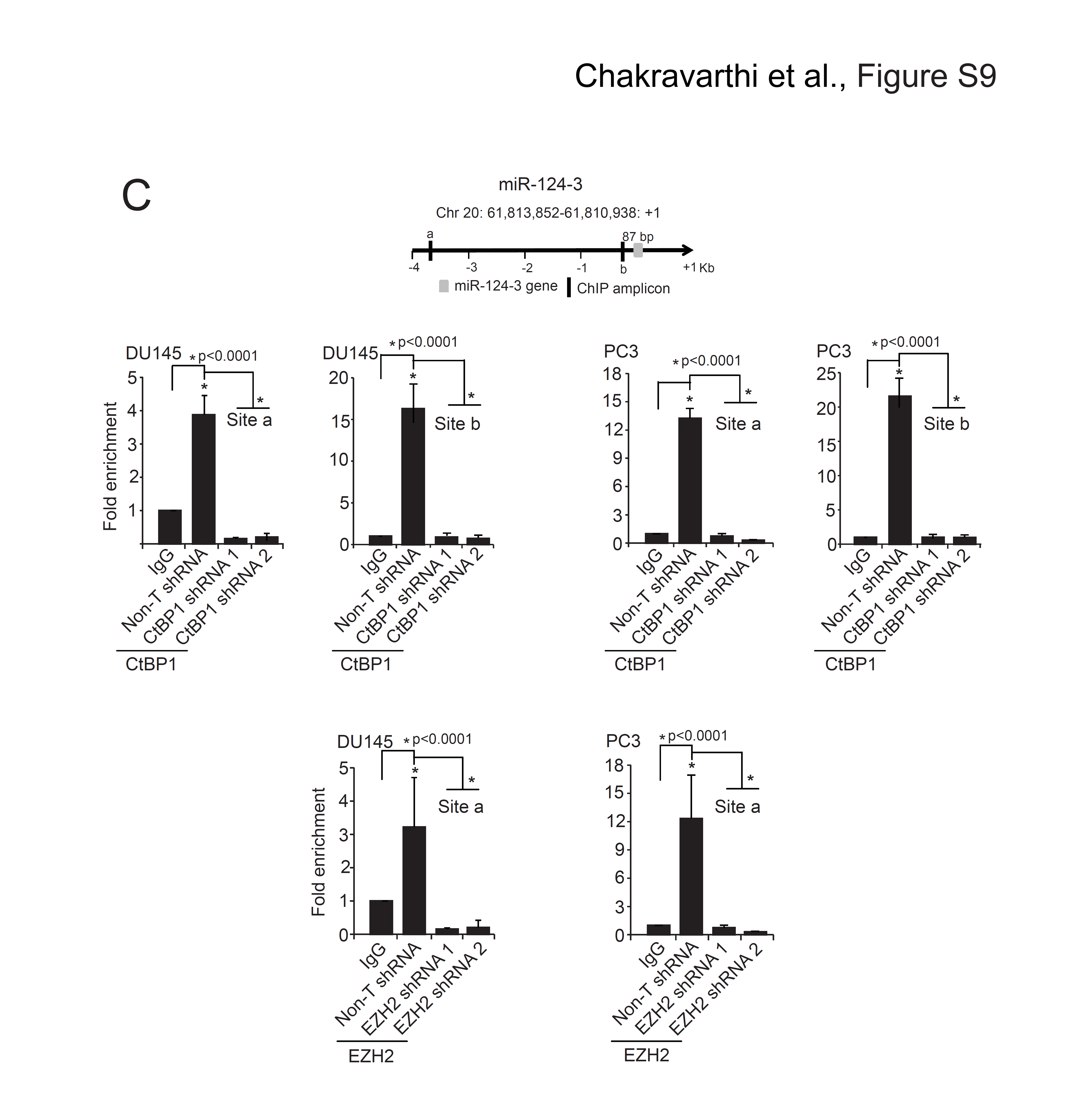


**Supplementary Figure S9, Related to Figure 5: EZH2 and CtBP1 occupy *miR-124-1*,*-2* and *-3* promoters.**

**(A, B, C)** Conventional Chromatin immunoprecipitation (ChIP)-PCR analysis of CtBP1 or EZH2 occupancy on *miR-124-1*, *-2* and *-3* promoters in DU145 and PC3 cells. ChIP was performed using antibodies against CtBP1 or EZH2 and a control IgG. Inset: Schematic representation of the *miR-124-1*, *-2* and *-3* genomic regions on chromosome 8 and 20 respectively, showing gene and amplicon positions. Error bars: n = 3, All bar graphs are shown with ± SEM.

###
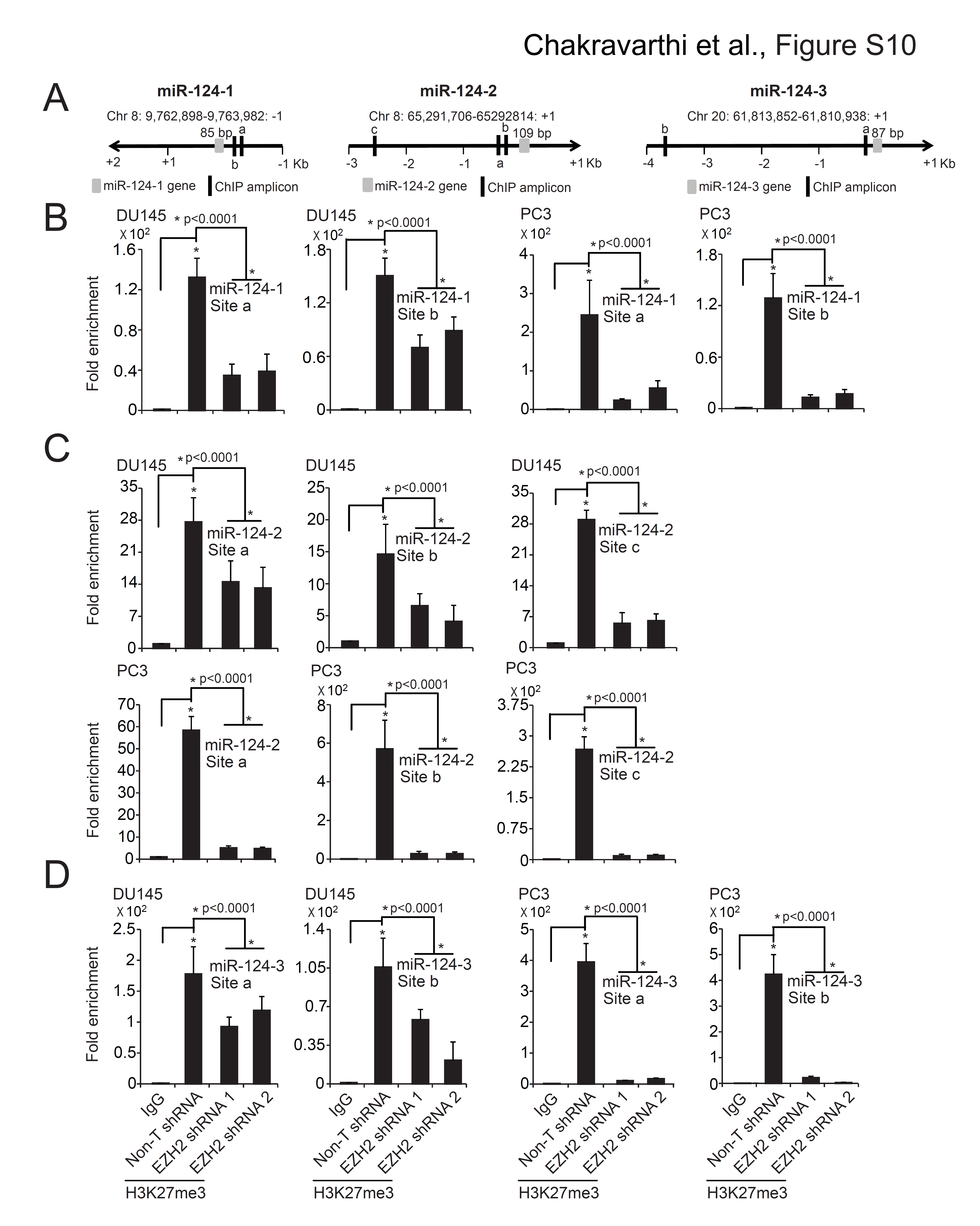


**Supplementary Figure S10, Related to Figure 5: The *miR-124* promoter is occupied with H3K27 trimethylation (H3K27me3).**

**(A)** Schematic representation of the *miR-124-1*, *miR-124-2* and *miR-124-3* genomic regions on chromosome 8 and 20 respectively, showing gene and amplicon positions. Conventional Chromatin immunoprecipitation (ChIP) PCR analysis for the level of H3K27 on **(B)** *miR-124-1*, **(C)** *-2* and **(D)** *-3* promoters in DU145 and PC3 cells. ChIP was performed using antibodies against H3K27 trimethylation mark and a control IgG. Error bars: n = 3, All bar graphs are shown with ± SEM.

###
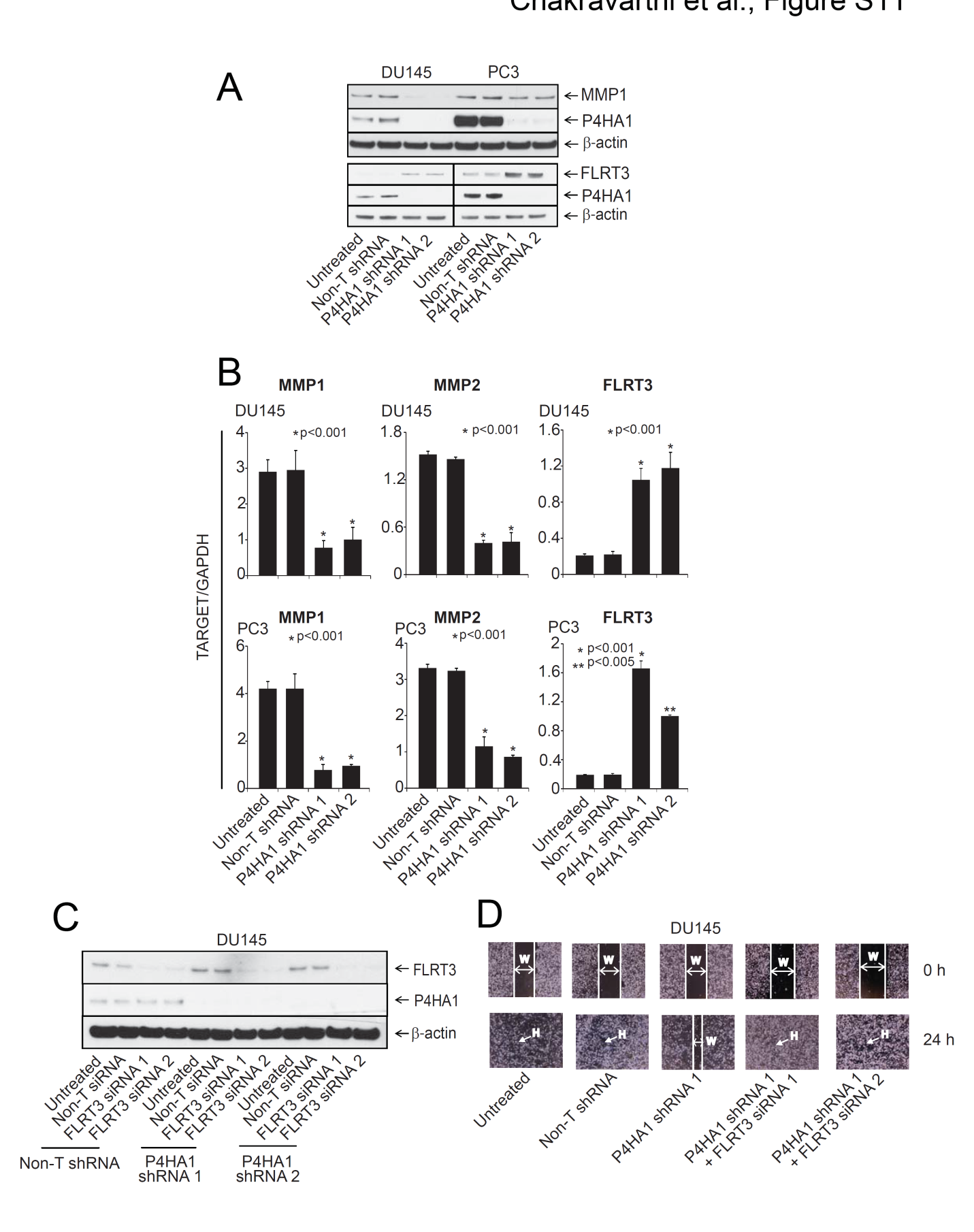


**Supplementary Figure S11,** **Related to Figures 6: P4HA1 knockdown modulates MMPs and FLRT3 involved in prostate cancer progression.**

**(A)** P4HA1 regulates expression of FLRT3 and MMP1. The expression of P4HA1, MMP1 and FLRT3 were measured in P4HA1 stable knockdown DU145 and PC3 cell lines by immunoblot analysis and, **(B)** *FLRT3*, *MMP1* and *MMP2* expression was measured in samples from **(A)** by qPCR. **(C)** Immunoblot analysis of FLRT3 and P4HA1 in transient FLRT3 knockdowns in stable DU145-P4HA1 knockdowns. **(D)** Wound healing assay in FLRT3 knockdown in stable DU145-P4HA1 knockdown cells. An artificial wound was created using a 0.2 ml pipette tip on a confluent monolayer of cells. Images were taken at 0 and 24 h after wound. The white lines show the margin of scratched area in which double headed arrow indicates scratch width (W) and white arrow indicates complete healing (H) of scratch wound. Two independent duplexes targeting FLRT3 were used. All bar graphs are shown with ± SEM.

###
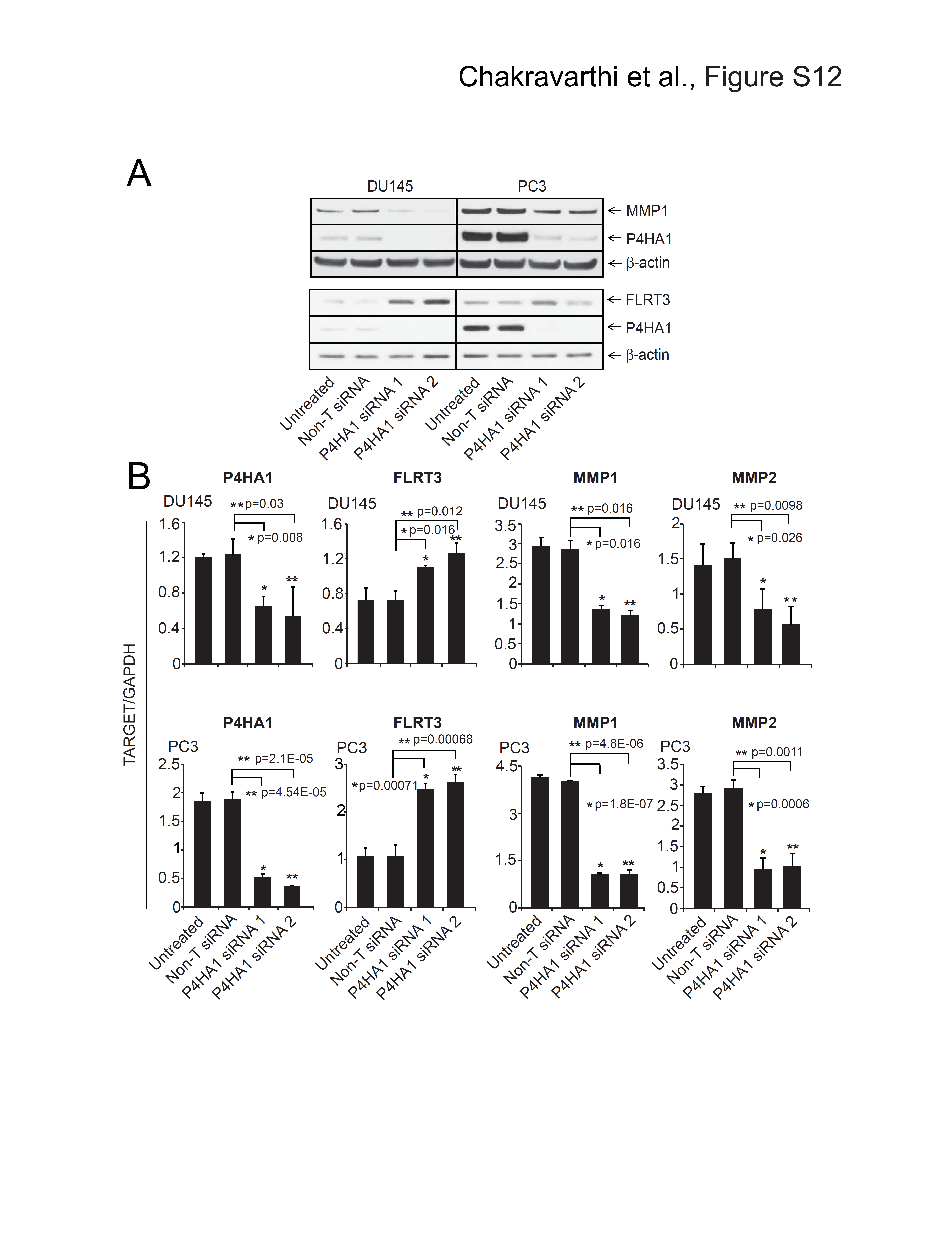


**Supplementary Figure S12, Related to Figures 6: Down-regulation of MMP1 and reactivation of FLRT3 by P4HA1 transient knockdown**

**(A)** P4HA1, MMP1 and FLRT3 protein expression by immunoblot analsyis. **(B)** qPCR analysis of *P4HA1*, *MMP1*, *MMP2* and *FLRT3* in transient P4HA1 knockdown DU145 and PC3 cells. All bar graphs are shown with ± SEM.

###
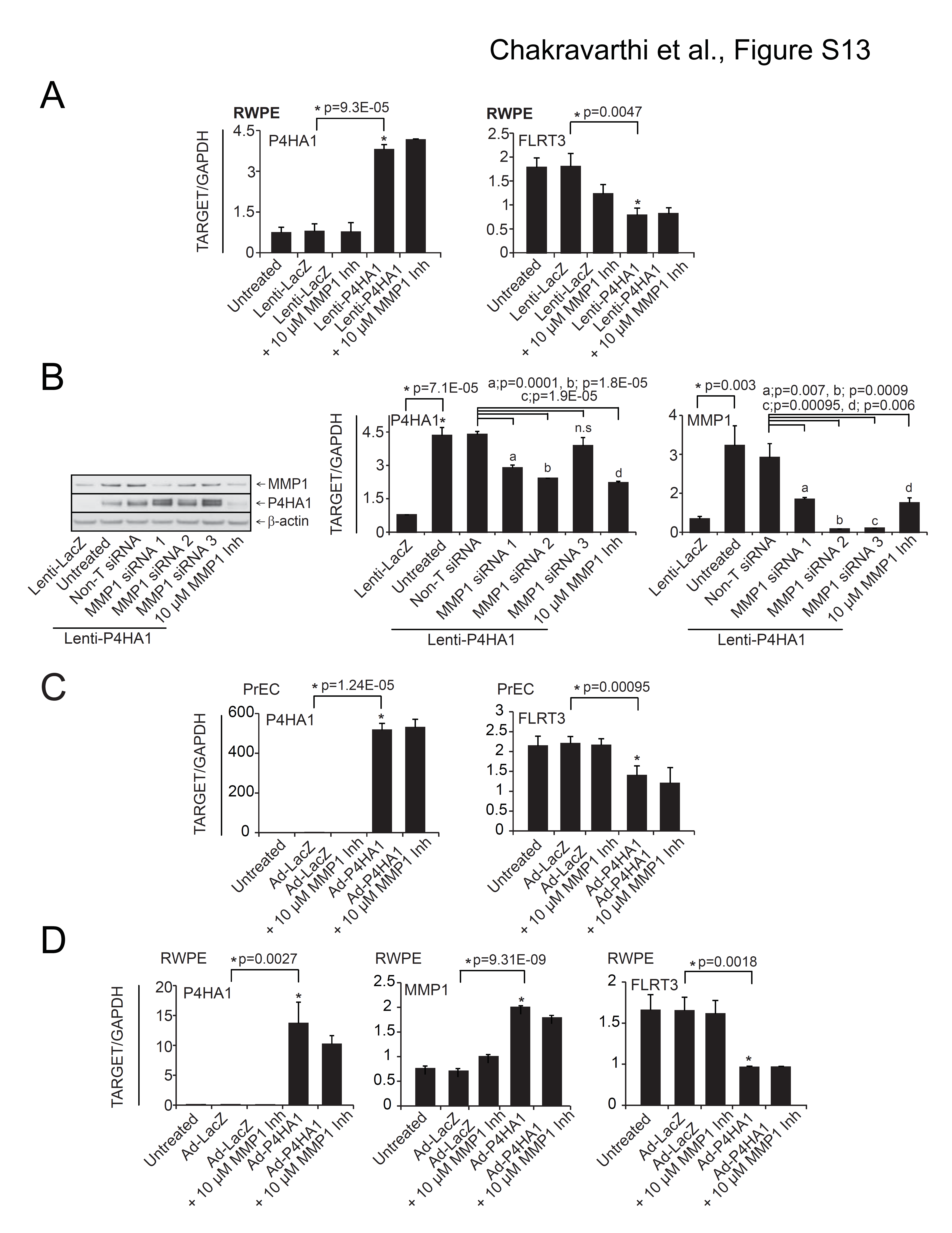


###

**Supplementary Figure S13, Related to Figure 6: Over-expression of P4HA1 modulates *MMP1* and *FLRT3* gene expression.**

Lentiviral over-expression of P4HA1. **(A)** qPCR analysis of *P4HA1* and *FLRT3* in RWPE cells stably over-expressing lacZ, P4HA1 alone or in the presence of 10 µM MMP1 inhibitor after 48 h. **(B)** Immunoblot and qPCR analyses of P4HA1 and MMP1 in RWPE cells stably over-expressing lacZ, P4HA1 alone, MMP1 siRNA treated or in the presence of 10 µM MMP1 inhibitor after 48 h; ns, not significant. **(C)** qPCR analysis of *P4HA1* and *FLRT3* in PrEC cells over-expressing adeno-lacZ, P4HA1 alone or in the presence of 10 µM MMP1 inhibitor after 48 h. **(D)** qPCR analysis of *P4HA1*, *MMP1* and *FLRT3* in RWPE cells over-expressing adeno-lacZ, P4HA1 alone or in the presence of 10 µM MMP1 inhibitor after 48 h. All bar graphs are shown with ± SEM.

###
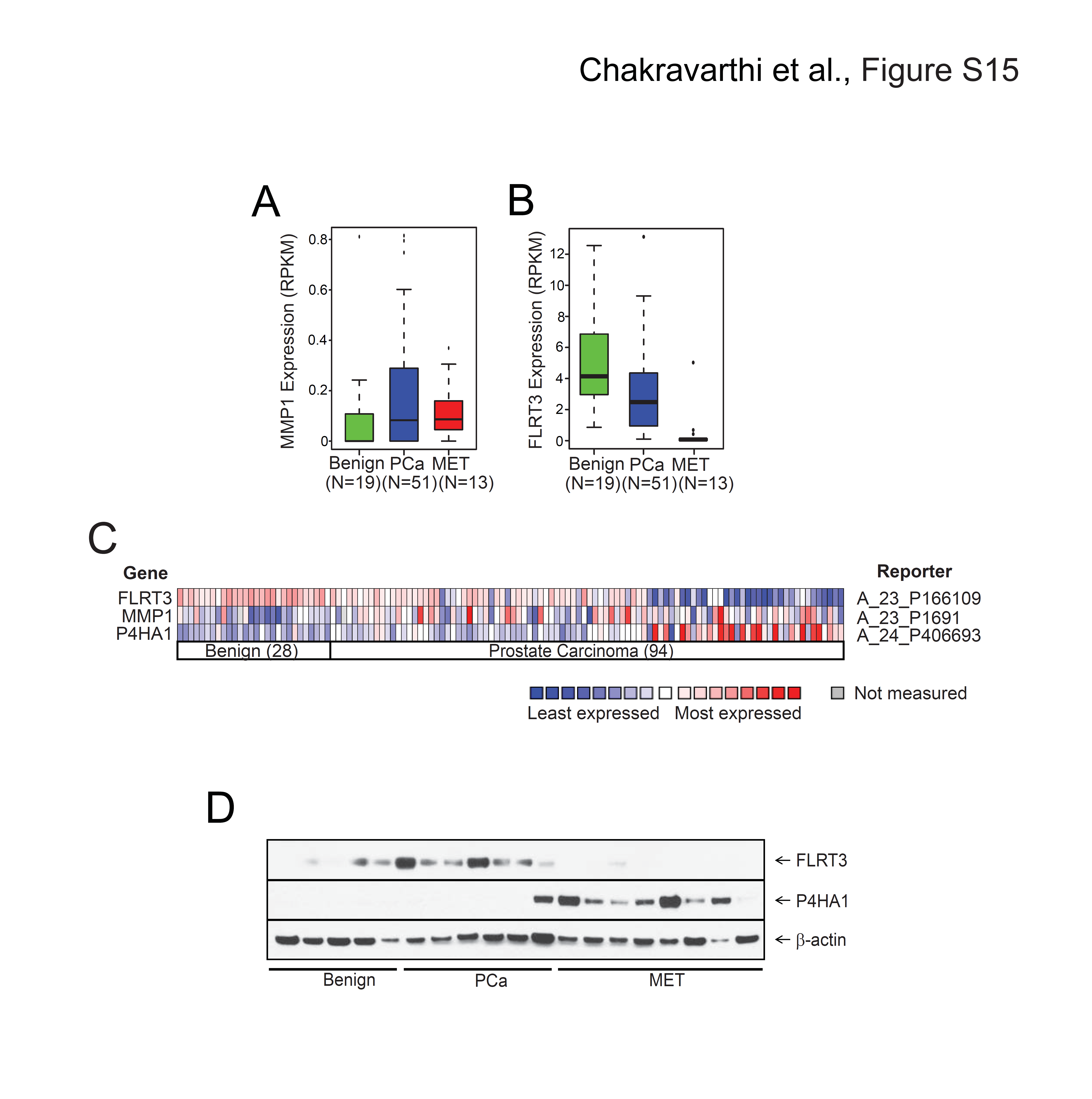


**Supplementary Figure S14, Related to Figures 6: Gene expression analysis of MMP1 and FLRT3 across prostate benign, carcinoma and metastatic tissues samples.**

**(A)** *MMP1* gene expression. **(B)** *FLRT3* gene expression. **(C)** Heat map of *P4HA1*, *MMP1* and *FLRT3* expression levels across prostate benign, carcinoma and metastatic samples. The data was retrieved from oncomine (Grasso et al., 2012). Blue and red color bars signify the lowest and highest levels of expression respectively. **(D)** P4HA1 and FLRT3 are inversely correlated in prostate cancer. Immunoblot analysis showing P4HA1 and FLRT3 across prostate benign, carcinoma and metastatic tissues protein lysates. Results are plotted as mean ± standard error.

**EXTENDED EXPERIMENTAL PROCEDURES**

**Chemicals and Reagents**

Cobalt(II) chloride hexahydrate (CoCl_2_, Cat. No. C8661) was purchased from Sigma Aldrich, USA. MMP1 inhibitor (tetrapeptidyl hydroxamic acid (FN-439)) was purchased from Calbiochem [Millipore, Billerica, MA (Cat. No. 444250)]. Recombinant human MMP1 protein (rhMMP1) was purchased from R & D systems [Minneapolis, MN (Cat. No. WBC024)].

### Benign and Tumor Tissues

In this study, we utilized tissues from clinically localized prostate cancer patients who underwent radical prostatectomy. Samples were also obtained from androgen-independent metastatic prostate cancer patients from a rapid autopsy program through the University of Michigan Prostate SPORE Tissue Core as described previously [[1](#_ENREF_1), [2](#_ENREF_2)]. The detailed clinical and pathological data are maintained in a secure relational database. This study was approved by the Institutional Review Board at the University of Michigan Medical School and informed consent was obtained from all subjects. Both radical prostatectomy series and the rapid autopsy program are part of the University of Michigan Prostate Cancer Specialized Program of Research Excellence Tissue Core.

**Processing of Microarray Data**

All microarrays were digitized using the Agilent Technologies Scanner G2505B and processed using the Agilent Feature Extraction software using the GE2-v5_95 protocol for two-color gene expression analysis. Log2 expression ratios (LogRatio column) and differential expression P-values (PValueLogRatio) were extracted from the files returned by the Agilent software. P-values were corrected for multiple hypothesis testing using the FDR method [[3](#_ENREF_3)] as implemented in R (p.adjust). Probe names (efg_agilent_wholegenome_4x44k_v1 identifiers) were translated into gene symbols via Ensembl [[4](#_ENREF_4)] and biomaRt [[5](#_ENREF_5)]. Differentially expressed genes were nominated using the following criteria: genes concordantly regulated in DU145 cells were required to show a log2 fold-change of 1.0, when up-regulated and -0.7, when down regulated; DU145 cells were required to show a log2 fold-change of 1.3 when up-regulated and -1.0 when down-regulated. Further, a less stringent supplementary set of differentially expressed genes in DU145 cells was defined as having a log2 fold-change greater than 1.0 in up-regulation or -0.65 in down-regulation. In addition, all probes were significant with an FDR-corrected p-value less than 0.001. All differentially expressed probes were arranged for visualization using hierarchical clustering using the Euclidean metric and displayed as heatmaps using Java TreeView [[6](#_ENREF_6)].

**Fluorescence *In situ* Hybridization (FISH) in cell lines and tumor samples**

­­

BAC clones were used to generate the locus specific FISH probe for *P4HA1* (RP11-41P9) and chromosome 10 control probe (RP11-351D16). Each BAC clone was grown in 200 ml LB medium containing 12.5μg/ml of chloramphenicol at 37°C for 14-16 hours with constant shaking. DNA was prepared using Qiagen- midiprep kit using Qiatip-100 according to the protocol provided by the manufacturer (Qiagen, USA). All FISH probes were prepared by nick translation labeling using modified nucleotides conjugated with biotin or digoxigenin utilizing biotin nick translation mix (11745824910, Roche, USA) for chromosome 10 control probe; digoxigenin nick translation mix (11745816910, Roche, USA) for *P4HA1* locus probe, respectively. Probe DNA was precipitated and dissolved in hybridization mixture containing 50% formamide, 2XSSC, 10% dextran sulphate, and 1% Denhardt’s solution. Approximately 200ng of each labeled probe was used for hybridization. Fluorescent signals were detected with Streptavidin Alexa fluor 594 (S-32356, Invitrogen, USA) and anti-digoxigenin fluorescein Fab fragments (11207741910, Roche, USA) for red and green fluorescence respectively. All clones were tested on normal human metaphase chromosomes to validate map position.

FISH scoring was performed by an experienced cytogeneticist (NP). *P4HA1* copy number was evaluated based on the ratio between control and locus specific probe. Copy numbers of 4 or greater in at least 15-20% of the cells in the tumor samples are considered amplified. Fluorescent images were captured using a high-resolution CCD camera controlled by ISIS image processing software (Metasystems, Germany).

**Immunohistochemistry**

Benign and prostate cancer tissues were obtained from the radical prostatectomy series at the University of Michigan and from the Rapid Autopsy Program, both part of the University of Michigan Prostate SPORE programs, through appropriate informed consent. Institutional Review Board approval was obtained to procure and analyze the tissues used in this study. Immunohistochemistry (IHC) was carried out to evaluate P4HA1 expression using rabbit polyclonal antibody against P4HA1 (PTG Labs [Chicago, IL], Cat# 12658-1-AP). IHC was performed using an automated protocol developed for the DISCOVERY XT automated slide staining system (Ventana Medical Systems, Inc.,) using Ultramap anti-rabbit HRP (Cat#760-4315,Ventana Medical Systems, Inc.,) and was detected using ChromoMap DAB (Cat#760-159, Ventana Medical Systems Inc.,). Hematoxylin II (Cat#790-2208 Ventana-Roche, Tucson, AZ, USA) was used as the counterstain. IHC staining was evaluated by Dr. Kunju (P.K.).

**Statistical Analyses (Related to Immunohistochemistry)**

For progression analysis, each core’s overall level of staining was summarized *via* a product score that was computed by multiplying staining percentage (0-100%) by staining intensity (2, 3, or 4, representing weak, moderate, or strong staining respectively). For patients with multiple cores on the TMA, staining score was obtained by taking the median product score across cores of the same type (tumor or normal) for that patient. Associations between staining and clinical parameters were calculated using Student’s t-test.

**Cell Proliferation Assays**

Cell proliferation was measured by cell counting. Stable, transient P4HA1 knock down, miR-124 treated or P4HA1-stably over-expressing cells were plated at 5,000 cells/well ( in 24-well plates (n = 3). Cells were harvested and counted at indicated time points by Coulter counter (Beckman Coulter, Fullerton, CA). Untreated, non-targeting siRNA, shRNA, miRNA or lacZ treated cells served as controls.

**Wound healing Assay**

DU145, PC3 control shRNA or P4HA1 stable knock down, RWPE-P4HA1 and -lacZ cells were seeded in 6-well plates in growth medium containing 10% fetal bovine serum and puromycin (10 µg/ml) for DU145 and PC3 or keratinocyte-Serum free medium (K-SFM) with supplements + blasticidin (3.5 µg/ml) for RWPE, and then allowed to grow to confluent monolayer. For DU145 and PC3, the cells were serum starved for 12 h and replenished with 10% FBS-RPMI medium. For RWPE, the stable cells were starved for 12 h with K-SFM without supplements. The wound-induced migration was triggered by scraping the cells with a pipette tip, washed with D-PBS and replenished with respective medium. The wound was imaged immediately (0 h) and at various time points as indicated with an inverted phase-contrast microscope with a 4X objective.

**miR Reporter Luciferase Assays**

Wild-type or mutant 3’-UTR (untranslated region) of P4HA1 were cloned into the pMIR-REPORT™ miRNA Expression Reporter Vector (Life Technologies, USA). HEK293 cells were co-transfected with pre-miR-124 or controls and WT or mutant 3’-UTR-luc as well as pRL-TK vector as internal control for luciferase activity. 48 hours post-transfection the cells were lysed and luciferase assays conducted using the dual luciferase assay system (Promega, Madison, WI). Each experiment was performed in triplicate.

**Induction of Hypoxia with CoCl_2_**

To examine the effect of hypoxia-mimetic agent CoCl_2_ on the expression of HIF1α, CtBP1, EZH2, P4HA1, miR-101,122, 124 and 499a, RWPE cells were plated into six-well plates (1 x 10­^5^ cells per well). After overnight in culture, each sample well was stimulated for indicated time intervals with 50 or 100 μM CoCl_2_ or solvent vehicle only as described earlier [[7](#_ENREF_7)] and then lysed in NP-40 lysis buffer for protein extraction or QIAzol® Lysis Reagent (Qiagen, USA) for RNA isolation. Similarly DU145, PC3 and PrEC cells were treated with 100 μM of CoCl_2_ for indicated time intervals.

**Chicken Chorio-Allantoic Membrane (CAM) assay**

The CAM assay for local cell invasion, intravasation, metastasis and tumor (or xenograft) formation was performed as previously described [[8-10](#_ENREF_8)]. Briefly, fertilized eggs were incubated in a rotary humidified incubator at 38°C for 10 days. CAM was dropped by making two holes, one through the eggshell into the air sac and a second hole near the allantoic vein that penetrates the eggshell membrane but not the CAM. Subsequently a cutoff wheel (Dremel) was used to cut a 1 cm^2^ window to expose the underlying CAM near the allantoic vein. Approximately 2 million cells in 50 μl of media were implanted in each egg, windows were sealed and the eggs were returned to the incubator. After 3 days, lower CAM was harvested and extra-embryonic tumors were isolated and weighed. For metastasis assay, the embryonic livers were harvested on day 18 of embryonic growth and analyzed for the presence of tumor cells by quantitative human Alu-specific PCR. Genomic DNA from lower CAM and livers were prepared using Puregene DNA purification system (Qiagen USA) and quantification of human-Alu was performed as described [[10](#_ENREF_10)]. An average of 8 eggs per group was used in each experiment.

**Statistical analysis**

### To determine significance differences between two groups, Student’s two-tail t test was used for all experiments except for microarray, p-values <0.05 considered significant.

**TABLES, Related to Methods**

**Table S1.** List of antibodies used in this study, Related to the Materials and methods.

| **Antibody** | **Application** | **Dilution** | **Supplier** | **Cat. No.** |
| --- | --- | --- | --- | --- |
| **P4HA1** | **IB,IHC,TMA** | IB, 1:1000  TMA and IHC, 1:100 | PTG Labs [Chicago, IL] | 12658-1-AP |
| **MMP1** | **IB** | IB, 1:1000 | EMD Millipore [Billerica, MA] | AB6002 |
| **FLRT3** | **IB** | IB, 1:1000 | Abcam [Cambridge, MA] | ab97267 |
| **HIF1α** | **IB, ChIP** | IB, 1:500-1000  ChIP, 1:100 | GeneTex [Irvine, CA] | GTX127309 |
| **CtBP1** | **IB, ChIP** | IB, 1:1000  ChIP, 1:100 | Sigma [St Louis, MO] | C8741 |
| **EZH2** | **IB, ChIP** | IB, 1:1000  ChIP, 1:100 | Cell Signaling Technology [Danvers, MA] | 5246 |
| **CALR** | **IB** | IB, 1:1000 | BD Transduction Laboratories [San Jose, CA] | 612136 |
| **Flag** | **IB** | IB, 1:500-1000 | Cell Signaling Technology [Denvers, MA] | 2368 |
| **β-actin** | **IB** | IB, 1:25000 | Sigma [St Louis, MO] | A5316 |
| **GAPDH** | **IB** | IB, 1:1000 | Abcam [Cambridge, MA] | ab8245 |
| **Histone H3K27me3** | **IB, ChIP** | IB, 1:1000  ChIP, 1:100 | Cell Signaling Technology [Denvers, MA] | 9756 |
| **Anti-Rabbit IgG HRP** | **IB** | IB, 1:5000 | GE Healthcare Bio-Sciences [Piscataway, NJ] | PI32460 |
| **Anti-Mouse IgG HRP** | **IB** | IB, 1:5000 | GE Healthcare Bio-Sciences [Piscataway, NJ] | 32430 |

**Table S2.** QPCR Primer sequences used, Related to the Materials and methods.

| **Gene Name** | **Forward primer** | | **Reverse primer** |
| --- | --- | --- | --- |
| **P4HA1** | GGCAGCCAAAGCTCTGTTAC | | AAAGCAGTCCTCAGCCGTTA |
| **CtBP1** | TCACAGGCCGGATCCCAGACAG | | GGTACCTATAGGCAGCCCCATTGAGC |
| **EZH2** | TGCAGTTGCTTCAGTACCCATAAT | | ATCCCCGTGTACTTTCCCATCATAAT |
| **P4HA2** | GGGAAGGTGACTACCGAACA | | CCTCGTTCATGGAACCACTT |
| **MMP1** | AGGTCTCTGAGGGTCAAGCA | | CTGGTTGAAAAGCATGAGCA |
| **MMP2** | AGCTCCCGGAAAAGATTGAT | | GGTGCTGGCTGAGTAGATCC |
| **FLRT3** | CCTGGAGCATCTTCCTCATC | | AGCGACACACAGATGGACAG |
| **HIF1α** | TGCTCATCAGTTGCCACTTC | | TCCTCACACGCAAATAGCTG |
| **Human Alu** | GTCAGGAGATCGAGACCATCCT | | AGTGGCGCAATCTCGGC |
| **Human Alu Taqman probe** | | 5′-6-FAM-AGCTACTCGGGAGGCTGAGGCAGGA-TAMRA-3′ | |

**Table S3.** SiRNA sequences used, Related to the Materials and methods.

| **Gene name** | **Supplier (Thermo Scientific)** | **Sequence** |
| --- | --- | --- |
| **P4HA1** | siRNA 1 (J-004275-07-0020)  siRNA 2 (J-004275-08-0020) | GGAAUUACAGGUAGCAAAU  GAUAAAGUCUCUGUUCUAG |
| **FLRT3** | **Supplier (Qiagen)** | **Target Sequence** |
|  | siRNA 1 (S103079692)  siRNA 2 (S103079692) | CCCTTCGAATGTACAACCCTA  CGCTTGGATGATAATCGCATA |
| **MMP1** | **Supplier (Thermo Scientific)** | **Sequence** |
|  | siRNA 1 (D-005951-02)  siRNA 2 (D-005951-03)  siRNA 3 (D-005951-04) | GGAGGUAUGAUGAAUAUAA  GAUGAAAGGUGGACCAACA  ACAGUAAGCUAACCUUUGA |

**Table S4.** QPCR Primer sequences used for HIF1α chromatin immunoprecipitation, Related to the Materials and methods.

| **Gene Name** | **Primer Name** | **Forward primer** | **Reverse primer** |
| --- | --- | --- | --- |
| **P4HA1** | P4HA1 Site a  P4HA1 Site b | GGAGGCAAACTGAACAGGAG  CTCCCACGTTCCTCTTCTCA | GTAAGCGGGAGTGGGATGTA  TCCTGTTCAGTTTGCCTCCT |
| **GLUT-1** | GLUT-1 | CTGCTCACTCATTCGTGCAT | CTGTGAGCACCTTGAGGACA |
| **VEGFA** | VEGFA | TGCCCCCTTCAATATTCCTA | GAGGAGGGAGCAGGAAAGT |

**Table S5.** QPCR Primer sequences used for HIF1α chromatin immunoprecipitation, Related to the Materials and methods.

| **Gene Name** | **Primer Name** | **Forward primer** | **Reverse primer** |
| --- | --- | --- | --- |
| **miR-124-1** | miR-124-1 Site a  miR-124-1 Site b | CCCACTCCGTCTGCCTAAG  CGAGCTCTCCCACTTCTCAC | CTGACTCCACAGCTCCCTTC  CACCAGCACACGTCATTCTC |
| **miR-124-2** | miR-124-2 Site a  miR-124-2 Site b ­­  miR-124-2 Site c | CTTGAGAAGAATATGCAATGACA  CTTCTGCGGCTCTTTGGTT  ACCAGAGGGGTAATTAACTTGGA | GGACGAATGTGCCTTTCAA  GGATTAATGGCTCGCTCCAG  TGTTCCGTGGAAGTGTAGACG |
| **miR-124-3** | miR-124-3 Site a  miR-124-3 Site b | CCAGACAGGAGACGCGAAC  GTTTCCTTGGGTCTCCGTGT | ACACTCCAGCCCCTCACAT  CCGAGGTGTCCTTGAGAACT |

**Table S6.** QPCR Primer sequences used for EZH2 and CtBP1 chromatin immunoprecipitation, Related to the Materials and methods.

| **Gene Name** | **Primer Name** | **Forward primer** | **Reverse primer** |
| --- | --- | --- | --- |
| **miR-124-1** | miR-124-1 Site a  miR-124-1 Site b | CTTTGGAAGACGTCGCTGTT  CCCCTTATTCATCTGCCTGT | GAGGAAGGGACCACAGCAT  CACACAAGGTCTTCGGCTTT |
| **miR-124-2** | miR-124-2 Site a  miR-124-2 Site b  miR-124-2 Site c | TGTGCAGGTTGCTGGAATTA  GTGTGCTGTAAATGGCATGG  GACAGGAGCTGGGCTTATGA | GACCTCACCATGGTCCTGAT  CAGCTCCTGTCTCTGCTCATC  AGACCCACTGCGATTACCAC |
| **miR-124-3** | miR-124-3 Site a  miR-124-3 Site b | GGAGATGTTTGGGGTCACAG  ATCCGTCTTCGCGATTCC | GGTGGTCAAAGGAGCTCAGA  CTTTCCGCGGCAGAGAGT |

**Table S7.** QPCR Primer sequences used for H3K27me3 chromatin immunoprecipitation, Related to the Materials and methods.

| **Gene Name** | **Primer Name** | **Forward primer** | **Reverse primer** |
| --- | --- | --- | --- |
| **miR-124-1** | miR-124-1 Site a  miR-124-1 Site b | CTTTGGAAGACGTCGCTGTT  GCGCCGCTTTTTATTTCTTT | GAGGAAGGGACCACAGCAT  CCTGAGGAAGGAAAGGAGGAAG |
| **miR-124-2** | miR-124-2 Site a  miR-124-2 Site b  miR-124-2 Site c | GACAGGAGCTGGGCTTATGA  GTGTGCTGTAAATGGCATGG  TGTGCAGGTTGCTGGAATTA | AGACCCACTGCGATTACCAC  CAGCTCCTGTCTCTGCTCATC  GACCTCACCATGGTCCTGAT |
| **miR-124-3** | miR-124-3 Site a  miR-124-3 Site b | GGAGAAGTGTGGGCTCCTC  ATCCGTCTTCGCGATTCC | GGTGGTCAAAGGAGCTCAGA  CTTTCCGCGGCAGAGAGT |

**SUPPLEMENTAL REFERENCES**

1. Tomlins SA, Laxman B, Dhanasekaran SM, Helgeson BE, Cao X, Morris DS, Menon A, Jing X, Cao Q, Han B, Yu J, Wang L, Montie JE, Rubin MA, Pienta KJ, Roulston D, et al. Distinct classes of chromosomal rearrangements create oncogenic ETS gene fusions in prostate cancer. Nature. 2007; 448(7153):595-599.

2. Tomlins SA, Rhodes DR, Perner S, Dhanasekaran SM, Mehra R, Sun XW, Varambally S, Cao X, Tchinda J, Kuefer R, Lee C, Montie JE, Shah RB, Pienta KJ, Rubin MA and Chinnaiyan AM. Recurrent fusion of TMPRSS2 and ETS transcription factor genes in prostate cancer. Science. 2005; 310(5748):644-648.

3. Benjamini Y and Hochberg Y. Controlling the False Discovery Rate: A Practical and Powerful Approach to Multiple Testing. J R Statist Soc B 1995; 57(1):289-300.

4. Hubbard T, Barker D, Birney E, Cameron G, Chen Y, Clark L, Cox T, Cuff J, Curwen V, Down T, Durbin R, Eyras E, Gilbert J, Hammond M, Huminiecki L, Kasprzyk A, et al. The Ensembl genome database project. Nucleic acids research. 2002; 30(1):38-41.

5. Durinck S, Moreau Y, Kasprzyk A, Davis S, De Moor B, Brazma A and Huber W. BioMart and Bioconductor: a powerful link between biological databases and microarray data analysis. Bioinformatics. 2005; 21(16):3439-3440.

6. Saldanha AJ. Java Treeview--extensible visualization of microarray data. Bioinformatics. 2004; 20(17):3246-3248.

7. Ivan M, Kondo K, Yang H, Kim W, Valiando J, Ohh M, Salic A, Asara JM, Lane WS and Kaelin WG, Jr. HIFalpha targeted for VHL-mediated destruction by proline hydroxylation: implications for O2 sensing. Science. 2001; 292(5516):464-468.

8. Asangani IA, Ateeq B, Cao Q, Dodson L, Pandhi M, Kunju LP, Mehra R, Lonigro RJ, Siddiqui J, Palanisamy N, Wu YM, Cao X, Kim JH, Zhao M, Qin ZS, Iyer MK, et al. Characterization of the EZH2-MMSET histone methyltransferase regulatory axis in cancer. Molecular cell. 2013; 49(1):80-93.

9. Brenner JC, Ateeq B, Li Y, Yocum AK, Cao Q, Asangani IA, Patel S, Wang X, Liang H, Yu J, Palanisamy N, Siddiqui J, Yan W, Cao X, Mehra R, Sabolch A, et al. Mechanistic rationale for inhibition of poly(ADP-ribose) polymerase in ETS gene fusion-positive prostate cancer. Cancer Cell. 2011; 19(5):664-678.

10. Wang R, Asangani IA, Chakravarthi BV, Ateeq B, Lonigro RJ, Cao Q, Mani RS, Camacho DF, McGregor N, Schumann TE, Jing X, Menawat R, Tomlins SA, Zheng H, Otte AP, Mehra R, et al. Role of transcriptional corepressor CtBP1 in prostate cancer progression. Neoplasia. 2012; 14(10):905-914.
